# Initial data analysis of the national German transplantation registry with a focus on kidney transplantation

**DOI:** 10.1186/s12874-026-03014-9

**Published:** 2026-10-15

**Authors:** Lukas Klein, Gunter Grieser, Carl-Ludwig Fischer-Fröhlich, Axel Rahmel, Henrik Stahl, Andreas Wienke, Antje Jahn-Eimermacher

**Affiliations:** 1 Present Address: Darmstadt University of Applied Sciences, Schöfferstraße 3, Darmstadt, 64295 Germany; 2 Present Address: Martin Luther University Halle-Wittenberg, Magdeburger Straße 8, Halle, 6112 Germany; 3 Deutsche Stiftung Organtransplantation, Deutschherrnufer 52, Frankfurt am Main, 60594 Germany

**Keywords:** Kidney transplantation, German transplantation registry, Initial data analysis, Missing data, Survival analysis

## Abstract

This study presents an Initial Data Analysis (IDA) of the German Transplantation Registry (TxReg) data for a better data understanding and to inform future data analyses. The IDA is focusing on data on first-time kidney-only transplantations in adult recipients from deceased donors between 2006 and 2016 and refers to data from 14,954 recipients and 9,964 donors across 25 tables. Investigated aspects include missing data patterns and structure, data consistency, and availability of event time data. Results show that missing data proportions vary widely, with some tables nearly complete while others have over 50% missing values. Missing data patterns are identified using a decision tree approach. An influx and outflux analysis demonstrates that some variables have high potential for imputing missing data, while others were less suitable for imputation. We identified 168 multi-sourced variables that are reported by multiple data providers in parallel leading to discrepancies for some variables but also providing opportunities for missing data imputation. Our findings on event time data demonstrate the importance of carefully selecting the variables used for event time analyses as results will strongly depend on this selection. In summary, our findings highlight the challenges when utilizing the TxReg data for research and provide recommendations for data preprocessing and analysis in future analyses.

**Supplementary Information:**

The online version contains supplementary material available at https://doi.org/10.1186/s12874-026-03014-9.

## Background

Since 2021, the *German Transplantation Registry (TxReg)* is the central archive for data on all solid-organ transplantations in Germany. It was established in the German transplantation law (Transplantationsgesetz, TPG) in 2016 [1] to collect data from the three data providers which coordinate the transplantation processes in Germany. The *Eurotransplant Foundation (ET)* is responsible for the allocation of organs in Germany, Austria, Belgium, Croatia, Hungary, Luxembourg, the Netherlands and Slovenia. In Germany, the *German Organ Transplantation Foundation (DSO)* is responsible for the coordination of organ donation and the *Institute for Quality Assurance and Transparency in Health Care (IQTIG)* is responsible for the quality assurance of the transplantation process. By national law, transplantation centers must report data of every transplantation procedure for the first three years of follow-up annually to IQTIG. For allocation, recipient data, as well as long-term follow-up data - especially in the context of retransplantation - must be stored at ET and transplantation centers. Therefore, transplantation data flows parallel through all three data providers to the TxReg.

The main goal of the TxReg is to improve the transparency and quality of the transplantation process by providing data for research and quality assurance [2]. However, the data has not been widely used for research purposes yet. At the time of writing, the official website of the registry only shows nine publications using the data. A potential reason for that might be the complexity of the data structure caused by the collection process from multiple data providers and potential quality issues of the data [3].

Before data are used to address research questions, initial data analysis (IDA) is essential to assess data quality, identify potential challenges, and evaluate suitability for subsequent analyses. The STRATOS initiative provides comprehensive guidance for IDA [4]. We used STRATOS IDA recommendations as a general orientation rather than a formal framework, focusing on aspects that are directly aligned with our objectives and deliverables: transparency and structured reporting, descriptive visualization of data structure, systematic assessment of missing data, and early use of IDA findings to inform subsequent analyses. In contrast to the typical STRATOS IDA setting, which assumes a specific research aim to guide IDA during a study, this article is designed as a general-purpose data resource intended to support a range of future research questions rather than a single predefined analysis.

Accordingly, this paper provides an overview of the TxReg data structure to facilitate future research use. We first analyze missing-data patterns and structure using flux analysis and a decision-tree approach. Flux analysis provides insight into the potential for missing-data imputation, whereas decision trees are used to identify predictors of missingness and to generate hypotheses about possible missing-data mechanisms. We then examine cross-provider consistency of information across variables and assess imputation potential. Finally, we provide guidance for using these data in time-to-event analyses of long-term outcomes, for example graft and patient survival.

We focus on the kidney data from the TxReg. Kidney failure, also known as end-stage kidney disease (ESKD), is a severe condition describing a complete loss of kidney function. It develops from chronic kidney disease (CKD) as the terminal stage. Common causes for CKD and ESKD include hypertension, other cardiovascular diseases, glomerular diseases, diabetes mellitus, cystic kidney diseases, urinary tract diseases, and congenital birth defects [5]. Diagnosis of ESKD is based on the glomerular filtration rate (GFR) [5].

Long-term treatment options for ESKD include dialysis and kidney transplantation [6]. Compared to dialysis, kidney transplant recipients show a higher quality of life and a lower mortality rate. These benefits are especially pronounced when a transplantation is performed before starting dialysis [7]. The availability of kidney transplantation as a treatment, however, is greatly limited by the number of available organs. In Germany in 2023, 2,617 registrations to the waiting list for a kidney transplantation were made [8]. In the same year, 2,122 kidney transplantations were performed. However, at the end of 2023, 10,454 patients were still on the waiting list [8]. Before a kidney transplantation can be performed, compatibility between the donor and recipient needs to be assured to prevent graft rejection and other complications. To optimize treatment effects both on a individual and a population level, an allocation algorithm is used to find recipients for a donor organ. Data from the TxReg allow e.g. important insight into the consequences of such organ allocation rules for certain patient groups [9].

When analyzing outcomes of kidney transplantation, there are different endpoints, which can be derived. When looking at graft failure, the following endpoints are common [10]:

1. **Patient survival**: Time from transplantation to death; individuals alive at last follow-up are censored at that time.
2. **Graft and patient survival**: Time from transplantation to irreversible graft failure (return to long-term dialysis or retransplantation) or death.
3. **Death-censored graft survival**: Time from transplantation to irreversible graft failure, with death while the graft is still functioning treated as a censoring event at death.

We start with notations and definitions including a description of the data structure of the TxReg data. We then describe the methods used for an analysis of missing data, data consistency and distribution of event time data. After presenting the results, we end with a discussion and conclusion.

## Methods

### Notations and definitions

The organization that is managing the TxReg data [11] internally uses four relational databases to collect, store, track and export data. For the export (to which we refer as the TxReg data), the data is provided in the form of a relational database, exported as comma-separated values (CSV) files. In the official documentation of the TxReg, three entities are described: transplantations, recipients and donors. Each of these entities in the database has a unique identifier provided by ET. In the Supplementary Table 1, Additional File 1, we provide an overview of the tables in the TxReg data and to which subject types they refer. Linkage between those entities is possible via the pseudonymized identifiers provided by ET [12].

When the data is provided to third parties for research purposes, it is anonymized. That includes encryption of identifiers and removal of personal information. Since certain dates could allow re-identification of patients, dates are provided as relative dates w.r.t. some undisclosed reference date. This allows to derive the time interval between transplantation and graft failure e.g. without exposing exact dates.

Each table contains columns and observations (rows). For the ease of reading we denote tables and columns by a prefix T_ and C_, respectively. For example, T_Transplantation.C_Destination refers to the column Destination in the table Transplantation. To allow international readability we use translated names for the columns, which are provided in the Supplementary Table 2, Additional File 1.

For a table $\textbf{X}$ (a CSV file in the export) we denote the number of observations and columns by $n_{\textbf{X}}$ and $p_{\textbf{X}}$, respectively.

To avoid ambiguity with transplantation centers, we refer to Eurotransplant (ET), the German Organ Transplantation Foundation (DSO), and the Institute for Quality Assurance and Transparency in Health Care (IQTIG) collectively as data providers (DPs). In formulas, the symbol $\text {DP}$ denotes the data provider contributing a column or observation.

All tables can contain missing values. However, especially for qualitative test results, explicit values like “not tested” or “unknown” exist, which we do not consider as missing values in the context of an IDA. We acknowledge that, depending on the variable and clinical context, such categories may in some settings be treated as missing values. In this analysis, however, we consistently treat them as informative categories rather than missing data.

Three data patterns (namely multi-sourced variables, relating observations and data providers and the mixing of cross-sectional and longitudinal data) play an important role for our IDA, we describe it them in the next subsections.

#### Multi-sourced variables

Each column $\textbf{X}_j$, $j=1,...,p_{\textbf{X}}$, of table $\textbf{X}$ is contributed by one of the data providers: ET, DSO or IQTIG. Supplementary Table 5, Additional File 1 shows to which tables which data provider contributed data. Thereby, not all columns may represent unique variables, due to the data collection process through three data providers. For example, the donor’s weight is stored in three columns, one from each data provider. We refer to such variables as “multi-sourced variables”. Their columns share the same name as a prefix and give the data provider identifier as a suffix. Multi-sourced variables can yield conflicting values but can also provide a chance to impute missing values.

To denote which data provider provides a column we use the following notation: Let ${\text {DP}}_{\textbf{X}j}$ be the data provider that contributes the $j\text {th}$ column of the table $\textbf{X}$. Let then $\mathcal {C}_\textbf{X}(\text {DP})$ be the set of all indices of columns contributed by data provider $\text {DP}\in \{\text {ET},\text {DSO}, \text {IQTIG}\}$ to table $\textbf{X}$. This set is defined as follows:

$$\begin{aligned} \mathcal {C}_\textbf{X}(\text {DP})= \{ j \in \{1, \ldots , p_{\textbf{X}}\} \;|\; {\text {DP}}_{\textbf{X}j}= \text {DP}\}. \end{aligned}$$

For example, in T_Recipient, $\mathcal {C}_\textbf{X}(\text {ET})$ contains all columns with suffix ET (e.g., EBasisGewichtWertET, EBasisGroesseWertET), while $\mathcal {C}_\textbf{X}(\text {IQTIG})$ contains the corresponding IQTIG-reported columns (e.g., EBasisGewichtWertIQTIG, EBasisGroesseWertIQTIG). A complete list of all multi-sourced variable pairs is provided in the digital supplementary material.

#### Relating observations to data providers

Not only variables, but also observations are contributed by all three data providers.

A data provider contributes an observation to the table if at least one of the columns contributed by the data provider has a non-missing value. Therefore we define $\mathcal {I}_\textbf{X}(\text {DP})$ to be the set of all indices of observations in table $\textbf{X}$ contributed by data provider $\text {DP}$ as follows:

$$\begin{aligned} \mathcal {I}_\textbf{X}(\text {DP})= \left\{ i \in \{1, \ldots , n_{\textbf{X}}\} \;|\; \exists j \in \mathcal {C}_\textbf{X}(\text {DP})\ \text {such that}\ \textbf{X}_{i,j}\ \textit{is not missing} \right\} . \end{aligned}$$

In practical terms, an observation can belong to more than one observation set $\mathcal {I}_\textbf{X}(\text {DP})$ (for different values of $\text {DP}$), depending on which provider-specific columns are non-missing in that observation. For example, in T_Transplantation, an observation with non-missing values in both ET and IQTIG columns would belong to both $\mathcal {I}_\textbf{X}(\text {ET})$ and $\mathcal {I}_\textbf{X}(\text {IQTIG})$. Therefore we consider provider-specific “sub-tables” within each TxReg table: for a given table, we analyze ET, DSO, and IQTIG contributions separately. Columns will always belong to a specific provider through $\mathcal {C}_\textbf{X}(\text {DP})$, while observations can belong to multiple providers through $\mathcal {I}_\textbf{X}(\text {DP})$. This directly reflects the TxReg data structure and avoids counting structurally absent entries as missing values.

#### Cross-sectional and longitudinal data

Some tables contain cross-sectional data, where each observation represents a single data entry, independent of the time of data collection. Other tables contain longitudinal data, where each observation represents a single data entry at a specified time point. The longitudinal data usually are complemented by a time identifier. Some tables contain both cross-sectional and longitudinal data. For example for the table T_Transplantation, ET contributes cross-sectional data, while IQTIG contributes longitudinal data. While ET uses an ET identifier for the identification of the transplantation entity, IQTIG uses in some cases the ET recipient identifier, in some cases the ET donor identifier and in some cases both identifiers. This leads to a situation where within the same table, multiple different classes of entities are present. Important to note here is that IQTIG does not report a time point for most observations. In these tables, column based data analysis for the IDA can lead to misleading results, because the number of observations is no longer meaningful in these tables. For example, the table T_Transplantation contains many more observations than transplantations as some transplantations are reported in more than one row. The correct number of transplantations however can be derived from the number of observations contributed by ET ($|\mathcal {I}_\textbf{X}(\text {ET}|$) with $\mathrm{X}=\mathrm{T}\backslash{\_}\mathrm{Transplantation}$. The number of observations for each entity is shown in Supplementary Table 6 to 8, Additional File 1. For this reason in our IDA we analyze the columns contributed by ET, DSO and IQTIG separately for each table.

### Target population

We are using the “legacy data” (Altdaten), comprising data collected between 2006 and 2016, i.e., before the TPG amendment 2016, which introduced the requirement for informed consent for data collection for research purposes.

The TxReg data primarily cover donors, transplantations, and recipients in Germany. Within Eurotransplant, however, a small number of postmortem donated organs from Germany are transplanted abroad, and conversely, a small number of organs donated abroad are transplanted in Germany. Geographically, the TxReg data therefore include donors from Germany, transplantations in Germany, and recipients in Germany, with minor cross-border exchanges.[12]

The initial data analysis is focusing on kidney transplantations where 1. the recipient is adult, 2. it is the first transplantation for this recipient and 3. the organ is from a deceased donor. The target population was constructed using the ET-reported recipients, donors, and transplantations in T_Recipient, T_Transplantation, T_Kidney_Waitlist, and T_Donor_PM. Recipients with multiple transplantations were identified and removed using T_Transplantation itself, but also using T_Kidney_Waitlist.C_KidneyTXCount. We only considered columns that had any data on this target population and removed duplicated columns, which led to the final set of 1123 columns used in the analyses. More details on the filtering process can be found in the supplementary material under Supplementary Information 1 and Supplementary Figure 1, Additional File 1. Our population consisted of 14,954 recipients and 9,964 donors across 25 tables.

### Missing data analysis

Missing values need to be accounted for in analysis and modelling. Many statistical and machine learning algorithms cannot handle missing values and require imputation, data deletion or encoding of missing values. Imputation often relies on the values of other columns, which are then used as predictors [13]. The decision on how to handle missing values is influenced by the number of missing values and the underlying structure. The following methods are applied to guide such decisions.

First, we analyze the proportion of missing values. $\mathbb{1}$ is the indicator function. By $M(\textbf{X}_j)$ and $R(\textbf{X}_j)$ we denote the number of missing and non-missing values for the $j\text {th}$ column of table $\textbf{X}$ contributed by data provider $\text {DP}$, respectively:

$$\begin{aligned} M(\textbf{X}_j)=\sum \limits _{i \in \mathcal {I}_\textbf{X}({\text {DP}}_{\textbf{X}j})}{ \mathbb{1}\left( \textbf{X}_{i,j}\ \textit{is missing}\right) } \end{aligned}$$

$$\begin{aligned} R(\textbf{X}_j)=\sum \limits _{i \in \mathcal {I}_\textbf{X}({\text {DP}}_{\textbf{X}j})}{ \mathbb{1}\left( \textbf{X}_{i,j}\ \textit{is not missing}\right) } \end{aligned}$$

Usually, the proportion of missing values is computed as the number of missing values divided by the total number of observations $n_{\textbf{X}}$. In our setting, this denominator is inappropriate, because aspects of the data representation induce structural missingness that does not reflect data quality. In addition to the mixed cross-sectional/longitudinal structure described, further representation issues occur: information from different data providers for the same event is usually stored in a single row with data-provider-specific columns, but in some instances it is stored across multiple rows. Consequently, some entries are systematically missing by design (representation/storage) rather than due to incomplete reporting.

Thus, instead of $n_{\textbf{X}}$ we use the number of observations contributed by data provider $\text {DP}$ ($|\mathcal {I}_\textbf{X}(\text {DP})|$). The proportion of missing values $PM(\textbf{X}_j)$ for column $j\text {th}$ in table $\textbf{X}$ can then be calculated as:

$$\begin{aligned} PM(\textbf{X}_j)=\frac{M(\textbf{X}_j) }{ |\mathcal {I}_\textbf{X}({\text {DP}}_{\textbf{X}j})|}. \end{aligned}$$

For ET-contributed columns in a table, the denominator is the number of observations with at least one non-missing ET value (not all rows of that table) In a complex dataset, not only the amount of missing data, but also the structure of the missing data is relevant for analysis. It can reveal mechanisms and potential sources for biases. Two methods are used to analyze the structures in the missing data. First, influx and outflux analysis according to van Buuren [14] is performed to identify patterns of missing data. Second, to identify potential causal mechanisms for missing data, a decision tree analysis to understand the structure of the missing data is conducted based on the work by Tierney et al. [15]. The first approach works vertically on the columns, while the second one operates horizontally on the observations.

#### Influx and outflux analysis

The influx is a measure describing the potential for imputing missing values in a column from not-missing values in other columns. Accordingly, the outflux describes the potential of not-missing values in a column to impute missing values in other columns.

When comparing two columns $\textbf{X}_{j}$ and $\textbf{X}_{k}$ of table $\textbf{X}$ from the same data provider ${\text {DP}}_{\textbf{X}j}={\text {DP}}_{\textbf{X}k}$, we can calculate different statistics based on pairwise comparison of the missingness within an observation. The proportion of usable cases $U(\textbf{X}_j,\textbf{X}_k)$ [14] between the *j*th and *k*th column in $\textbf{X}$ from the same data provider is the proportion of missing values of $\textbf{X}_{j}$ that could be imputed by the use of non-missing observations in $\textbf{X}_{k}$. It is defined as follows:

$$\begin{aligned} U(\textbf{X}_j,\textbf{X}_k) = \frac{\sum \nolimits _{i \in \mathcal {I}_\textbf{X}({\text {DP}}_{\textbf{X}j})}{ \mathbb{1}\left( \textbf{X}_{i,j} \textit{ is missing}\wedge \textbf{X}_{i,k} \textit{ is not missing}\right) }}{M(\textbf{X}_j)} \quad \text {for } {\text {DP}}_{\textbf{X}j}= {\text {DP}}_{\textbf{X}k} . \end{aligned}$$

This statistic can be used to check whether a column $\textbf{X}_{k}$ has the potential to be used for missing data imputation of column $\textbf{X}_{j}$. Influx $I(\textbf{X}_{j})$ and outflux $O(\textbf{X}_{j})$ of column $\textbf{X}_j$ are the application of the same approach to all columns from the same data provider of table $\textbf{X}$.

The influx is the proportion of missing value in any column $k \in \mathcal {C}_\textbf{X}({\text {DP}}_{\textbf{X}j})$ that could be imputed by the use of a non-missing observation in $\textbf{X}_{j}$. The outflux is the proportion of non-missing value in any column $k \in \mathcal {C}_\textbf{X}({\text {DP}}_{\textbf{X}j})$ that could be used to impute any missing value in $\textbf{X}_{j}$ [14].

$$\begin{aligned} I(\textbf{X}_{j})= \frac{\sum \nolimits _{k \in \mathcal {C}_\textbf{X}({\text {DP}}_{\textbf{X}j})}\sum \nolimits _{i \in \mathcal {I}_\textbf{X}({\text {DP}}_{\textbf{X}j})} \mathbb{1}\left( \textbf{X}_{i,j} \textit{ is missing}\wedge \textbf{X}_{i,k} \textit{ is not missing}\right) }{\sum \nolimits _{k \in \mathcal {C}_\textbf{X}({\text {DP}}_{\textbf{X}j})}R(\textbf{X}_k)} \end{aligned}$$

$$\begin{aligned} O(\textbf{X}_{j})= \frac{\sum \nolimits _{k \in \mathcal {C}_\textbf{X}({\text {DP}}_{\textbf{X}j})}\sum \nolimits _{i \in \mathcal {I}_\textbf{X}({\text {DP}}_{\textbf{X}j})} \mathbb{1}\left( \textbf{X}_{i,j} \textit{ is not missing}\wedge \textbf{X}_{i,k} \textit{ is missing}\right) }{\sum \nolimits _{k \in \mathcal {C}_\textbf{X}({\text {DP}}_{\textbf{X}j})}M(\textbf{X}_k)} \end{aligned}$$

These values depend on the number of missing values, therefore comparisons of influx and outflux are usually not reasonable. We visualize influx and outflux in a scatterplot. Only for T_Recip_Virology it was not possible to calculate the outflux, as there are no missing values in any column.

#### Missing data structure analysis

Tierney et al. [15] proposed a method to better understand the structure of missing data in that they investigate which columns in a table best predict the number of missing values per observation. For this, they proposed a regression tree. By analyzing the structure of the tree, it can be identified which columns are important predictors for the number of missing values per observation. This can help to understand the mechanisms of missing data and to identify potential sources of bias or improve downstream analyses.

The target variable for training the trees is the proportion of missing values per observation. We calculate trees separately by data provider for the reasons described. Thus, for a data provider $\text {DP}$ the target variable for observation $i \in \mathcal {I}_\textbf{X}(\text {DP})$ is calculated as:

$$\begin{aligned} OPM(\textbf{X},\text {DP},i) = \frac{\sum \nolimits _{j \in \mathcal {C}_\textbf{X}(\text {DP})} \mathbb{1}\left( \textbf{X}_{i,j} \textit{ is missing}\right) }{|\mathcal {C}_\textbf{X}(\text {DP})|} \end{aligned}$$

Each table was split into a training and a test set with a 70/30 split. The regression tree was trained on the training set and the accuracy of the prediction was evaluated on the test set using the root mean squared error (RMSE). The rpart [16] package was used for tree building, as it supports surrogate splits. Training used default parameters for variance splitting: a minimum of 20 observations for a split, a minimum of 7 observations in a leaf, a complexity parameter of 0.01, 5 maximum surrogates, majority vote if a surrogate split is not possible, 10 cross-validation folds, and a maximum depth of 30. For training, we only considered columns with at least two distinct values. Additionally, non-numeric columns were considered only if they have at most 250 distinct values.

Adjusted feature importance was calculated to order the columns by their importance for predicting the proportion of missing values per observation. The importance of a predictor in rpart is calculated by the sum of the goodness of split measures plus the adjusted goodness of split measures for surrogate splits [16].

### Data consistency

Beyond data completeness, data consistency represents a critical aspect for reliable analyses. In particular, the multi-sourced variables, present both analytical challenges and opportunities. While they cause collinearity that negatively affects analyses e.g. by increasing standard errors, imputation strategies could highly benefit from using this information: Missing values in one data provider’s column can potentially be imputed using values from another data provider’s corresponding column, thereby improving overall data completeness. We therefore first examine the potential of multi-sourced variables for imputation and second assess the degree of agreement between information reported by several data providers. These analyses can inform both decisions on data preprocessing and variable selection.

The proportion of usable cases $U(\textbf{X}_j,\textbf{X}_k)$ will be used to identify the potential of missing data imputation by the multi-sourced variables. To analyze the consistency and agreement of information between columns, Cramér’s V and the Pearson correlation coefficient are calculated. Cramér’s V is a measure of association between two categorical variables and is calculated from the chi-squared statistic. It ranges from 0 (no association) to 1 (perfect association). For two numerical variables, the absolute value of the Pearson correlation is calculated, which ranges from 0 (no linear association) to 1 (perfect linear association). The absolute value is used as we are interested in the strength of the association.

### Distribution of event time data

A major potential of registries like the TxReg is the ability to study long-term outcomes of solid organ transplantations, such as time-to-event outcomes (e.g., patient death, graft failure, or combinations of these) using survival analysis. We explore important information given in TxReg to enable such analyses. Thereby, we focus on event time analysis for the time from organ transplantation to patient death and/or organ failure, that is a common outcome used in research on transplantation strategies [17–19].

For event time analyses of graft and patient survival, the following information is needed: The date of organ transplantation, the date of patient death, the date of organ failure and the date of last contact. The latter is required to properly consider the time patients are at risk and observed for experiencing events in estimates of incidence rates and proportions. As already mentioned dates cannot be interpreted as calendar dates, instead dates will refer to relative dates only.

These four dates needed for event time analyses are provided by different data providers and are distributed across tables and columns making the extraction of these dates ambiguous. We will explore the use of different variables to derive these dates by visualizing their distributions with kernel density estimates and illustrating associations between variables by scatterplots.

All analyses and processing steps were implemented with Python, Snakemake [20] and the pandas [21, 22] package. Visualizations and further downstream analyses of results were implemented with R and the tidyverse [23] family of packages. A full list of packages and versions used is available in the Digital Supplementary material.

## Results

### Missing data analysis

**Fig. 1:**
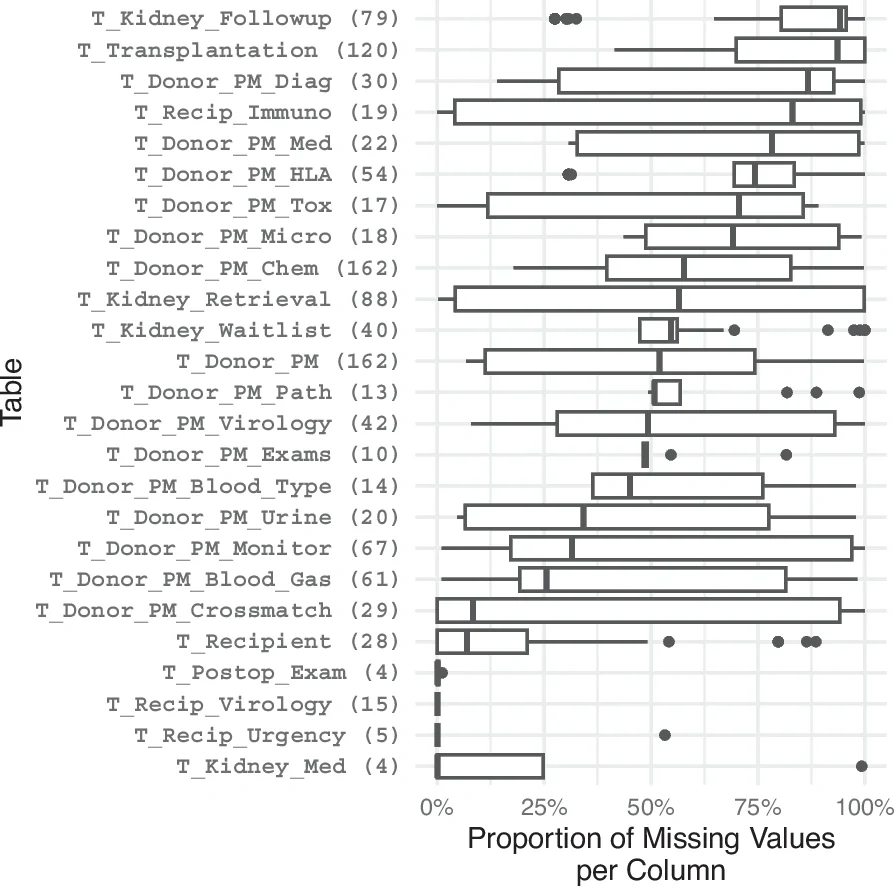
Boxplot of the proportion of missing values in each column across all tables

Figure 1 illustrates the proportion of missing data in each column of the tables $PM(\textbf{X}_j)$.

Our analysis reveals that T_Kidney_Med, T_Recip_Urgency, T_Recip_Virology and T_Postop_Exam are mostly complete, with only a few columns having missing values and their median proportion of missing values being close to 0%. For T_Recip_Virology this can be explained with the use of explicit missing value indicators like “not tested”. The other tables have a small number of nearly complete columns. Most of them are identifiers or time point information for longitudinal data.

Our analysis also identifies that the tables with columns with high proportions of missing values are T_Kidney_Followup, T_Transplantation, T_Donor_PM_Diag, T_Recip_Immuno and T_Donor_PM_Med. When looking at the data, all of these have in common that they contain longitudinal data. Within these tables, each observation refers to a specific event or lab test. For example, T_Kidney_Followup contains follow-up events, which can be either initial or recurrent follow-ups. T_Recip_Immuno contains lab tests for immunological parameters, such as HLA typing or antibody screening. Depending on which test or event is reported, different columns are used to report the data, implying that other columns are missing. This could explain the high proportions of missing observations per column in these tables.

**Fig. 2:**
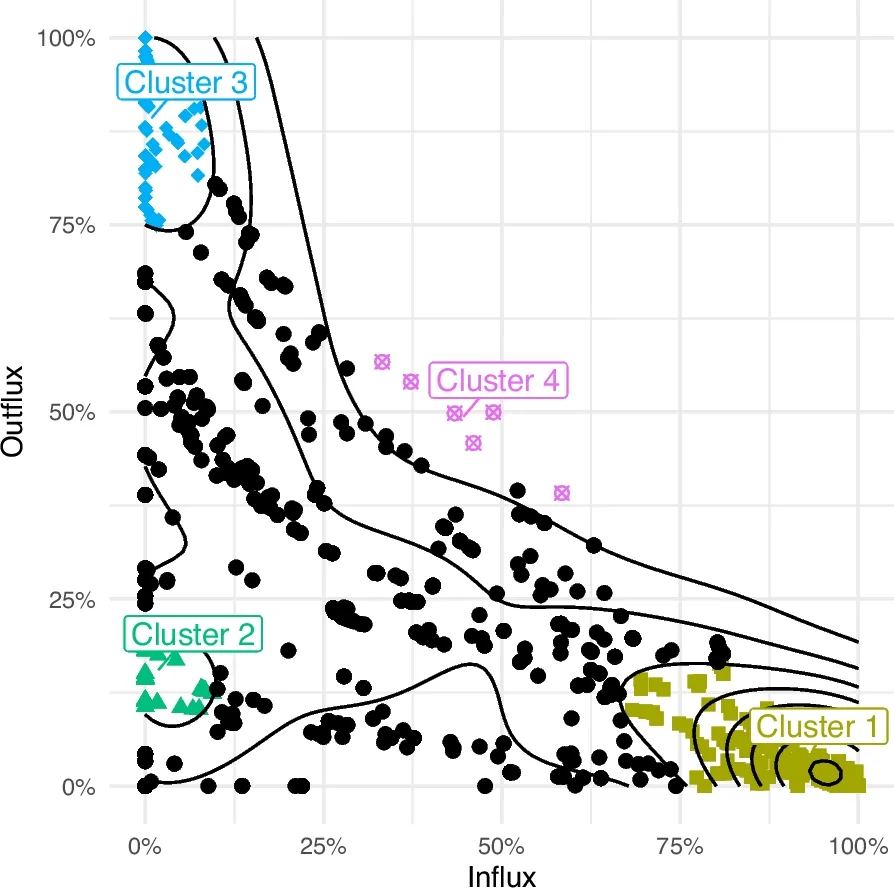
Flux analysis plot: Influx $I(\textbf{X}_{j})$ is shown on the x-axis and outflux $O(\textbf{X}_{j})$ on the y-axis. Contours of a two-dimensional density estimate are shown in the background

These findings show that simple ad-hoc imputation strategies like mean imputing e.g. should not be applied. We recommend the proper separation of the different types of observations (e. g. lab test types) before imputation. The following results on a missing data structure analysis can support researchers in defining a reasonable imputation strategy:

Good candidates for an imputation model are column pairs which have non-missing values co-occurring with missing values or vice versa. These two properties are measured with the outflux $O(\textbf{X}_{j})$ and influx $I(\textbf{X}_{j})$. The results are shown in Fig. 2.

Each column of each table is depicted as a point in Fig. 2. The contours of a density estimate are included to highlight clusters of columns. Based on a cluster analysis, we identified four clusters of interest.

- Cluster 1: Missing values in columns of this cluster may be easy to be imputed by other columns, but the column itself may be not very useful to impute other columns.
- Cluster 2: Missing values in columns of this cluster may by difficult to be imputed by other columns and the column itself maybe not very useful to impute other columns.
- Cluster 3: Missing values in columns of this cluster may be difficult to be imputed by other columns, but the column itself my be useful to impute other columns.
- Cluster 4 contains columns with both medium influx and outflux. These columns have many non-missing values co-occurring with missing values and vice versa. They might also be useful for imputation.

A list of the columns in clusters 3 and 4 is shown in Supplementary Table 3, Additional File 1.

It is important to note here that influx and outflux only provide first insights into the usability of a column for imputation without providing information how efficient an imputation finally will be. However, a pre-selection of columns based on the flux analysis can help to identify columns which are more likely to be useful for imputation.

Missing values might be caused by known or unknown mechanisms in the data collection and reporting process.

Table 1 shows columns which were important predictors for the proportion of missing values per observation $OPM(\textbf{X},\text {DP},i)$. The RMSE gives the prediction error on the test set. Because analyses were performed separately for each $\text {DP}$, results are given for each $\textbf{X}$ and $\text {DP}$. Columns with an adjusted importance above 50% were considered an important predictor and are shown in this table. Full results are available in Supplementary Table 4, Additional File 1.

**Table 1:** Important predictors for the proportion of missing values in an observation in each table and contributing data provider

Table (DP)	Test RMSE	Important Predictors
T_Recip_Immuno (ET)	0.029	C_ResultType, C_DiagCenter
T_Donor_PM_Diag (DSO)	0.033	C_DiagClass
T_Recip_Urgency (ET)	0.055	C_UrgeCode
T_Donor_PM_Crossmatch (DSO)	0.062	C_TXC, C_XMERG
T_Donor_PM_Med (DSO)	0.071	C_DosUnit
T_Transplantation (ET)	0.089	C_FolUpCenter, C_RegCenter, C_TXCenter
T_Kidney_Followup (ET)	0.102	C_FolUpCenter, C_FolUpType
T_Donor_PM_Blood_Type (DSO)	0.115	C_DiagType
T_Donor_PM_Monitor (DSO)	0.168	C_ArrestDur

The decision tree analysis revealed patterns in the missing data structure across all tables shown in Table 1. A clear finding emerged: columns identifying observation types (e.g., test type, diagnosis class, urgency code) and centers were able to predict missing data patterns.

Type identifiers were the most important predictors in five of the nine table-data-provider pairs. For example, in T_Donor_PM_Diag, the diagnosis class C_DiagClass (identifying one of ten different diagnosis types) was the primary predictor. Similarly, in T_Recip_Immuno, the lab result type C_ResultType was the strongest predictor, reflecting that different laboratory tests use different sets of columns. In T_Recip_Urgency, the urgency code C_UrgeCode determined which additional urgency-specific columns were completed. This highlights how important it is to separate the different types of observations (e.g., lab test types) before imputation or analysis.

Center identifiers were also prominent predictors, appearing in three table-data-provider pairs. In T_Recip_Immuno, the diagnostic center C_DiagCenter was the second most important predictor alongside the test type, indicating that different centers report different sets of results. For T_Transplantation and T_Kidney_Followup, various center columns (follow-up center, registration center, transplant center) predicted missingness, suggesting center-specific reporting practices. Here, the centers might perform different types of follow-ups or have varying protocols for data collection. However, center identifiers in the exported dataset are anonymized/pseudonymized (in accordance with §15g TPG), so linkage to real transplant centers is not possible under the current data access agreement [12]. This should be considered when interpreting center-level differences in missingness patterns.

Notable exceptions were the dosage unit in T_Donor_PM_Med and cardiac arrest duration in T_Donor_PM_Monitor, which may serve as surrogates for medication or monitoring type information. The prediction varied, test RMSE values in the shown results ranged from 0.029 to 0.168, demonstrating that missing value patterns can be reliably predicted using these structural variables.

### Data consistency

**Fig. 3:**
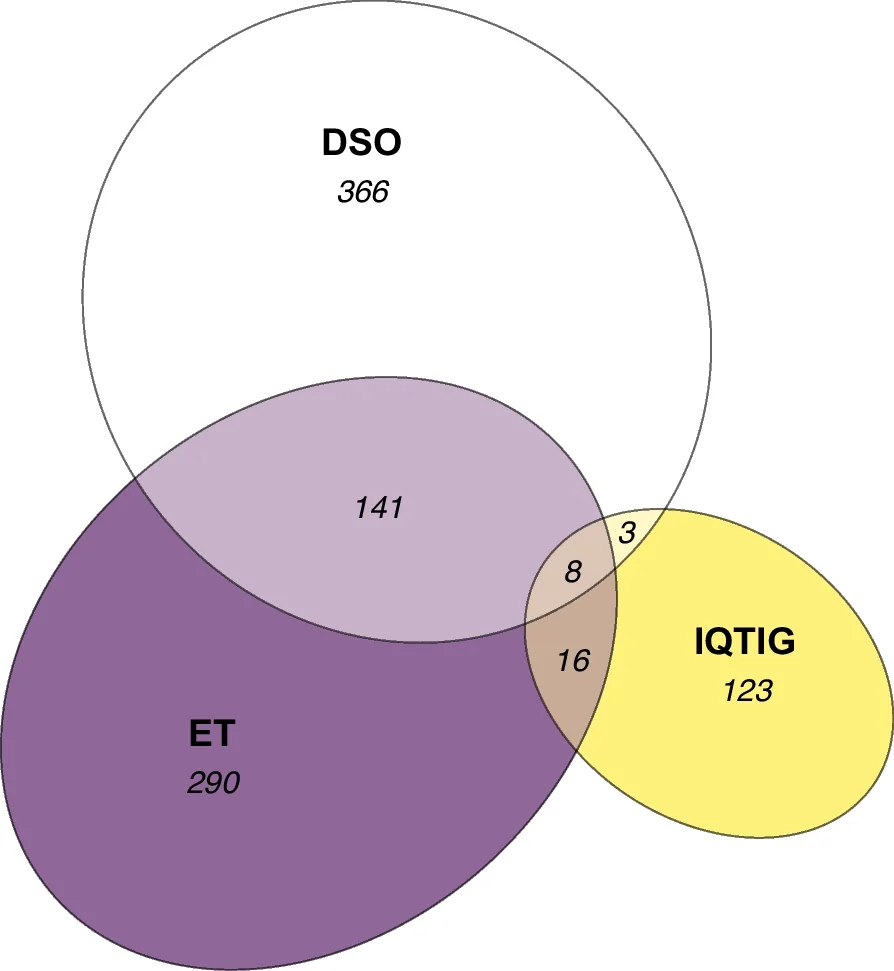
Number of columns contributed by each data provider. Overlaps are multi-sourced variables

First, we analyzed the multi-sourced variables. Figure 3 visualizes the number of columns that represent these multi-sourced variables, grouped by the data provider.

Our analysis identified 168 multi-sourced variables across all tables. Of those, 141 are provided by DSO and ET, while only 8 variables are provided by all three data providers (DSO, ET, and IQTIG).

**Fig. 4:**
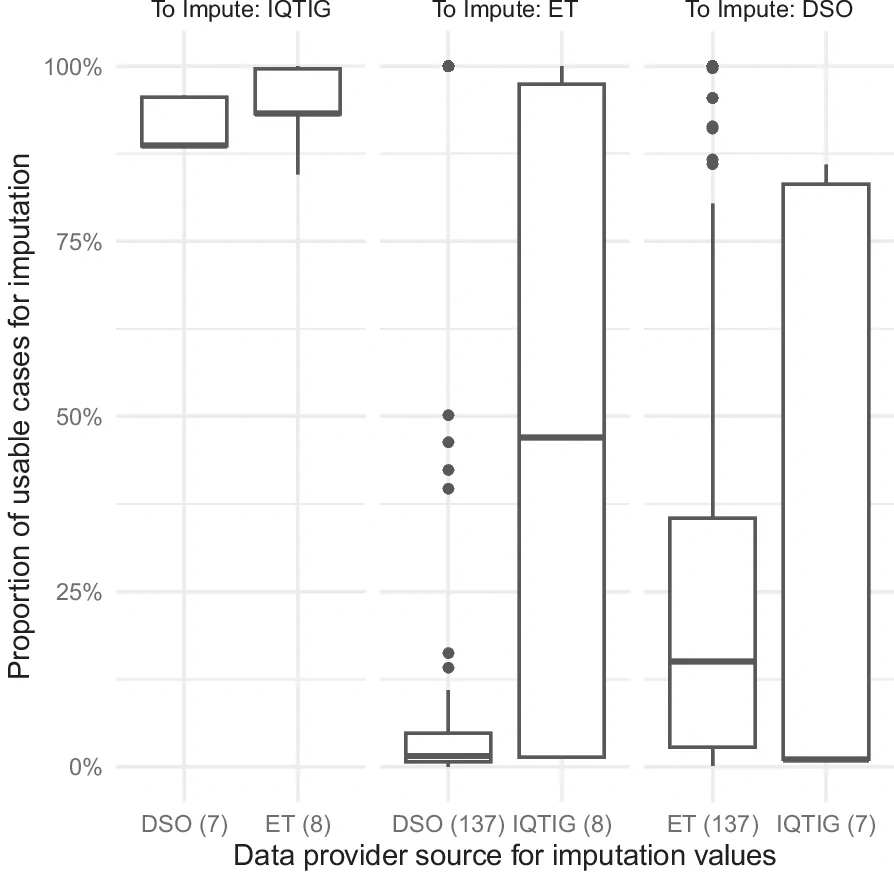
Boxplots of the usability of columns in multi-sourced variables for imputation

Next, we investigate the potential of the multi-sourced variables for cross–data-provider imputation by analysing the proportion of usable cases $U(\textbf{X}_j,\textbf{X}_k)$. Figure 4 displays the distribution of $U(\textbf{X}_j,\textbf{X}_k)$ values across pairs of multi-sourced variables. Only pairs are shown where both data providers reported a value for at least one observation. In this visualization, the y-axis represents $U(\textbf{X}_j,\textbf{X}_k)$, the x-axis shows the target data provider for imputation, and the panel headers indicate the source data provider providing the imputation values. As an example, for missing values in IQTIG columns, the median proportion of usable cases for imputation when using corresponding DSO columns as sources was 89%, indicating high imputation potential.

The results reveal distinct patterns of imputation utility across data-provider combinations. For missing values in multi-sourced IQTIG columns, approximately 80% of cases had corresponding non-missing values in either DSO or ET columns, indicating these sources provide substantial imputation opportunities for IQTIG data. Conversely, imputation of multi-sourced ET columns using DSO sources showed limited potential, with only few column pairs exhibiting proportions of usable cases above 25%. The observed outliers reaching 100% usability corresponded to entry dates and ET identifier columns.

IQTIG columns provided limited utility for imputing missing ET or DSO values. The potential for imputting multi-sourced DSO columns using ET sources is also limited (median 15% usable cases) but still higher as the reverse scenario of imputing ET columns from DSO sources (median 2% usable cases). Based on these findings, we recommend prioritizing ET and DSO columns for primary analysis while utilizing IQTIG columns primarily as sources for imputing missing values in ET and DSO datasets.

This imputation strategy is based on the assumption that all data providers provide the same kind of information and thus give consistent results. In the following we show inter–data-provider agreements for all pairs of columns within multi-sourced variables. For 37 out of 184 pairs, no association could be calculated due to constant data, no non-missing values occurring for the same observation or data type mismatches.

Of the 147 column pairs for which we could calculate association measures, 139 pairs showed a high agreement with a measured association of 0.9 and above. The 8 pairs that exhibit associations below 0.9 and thus some disagreement between data providers are shown in Table 2. For example, the recipient’s height columns from the DSO and ET in T_Recipient show an absolute value of the Pearson correlation coefficient of only 0.01, that indicates that DSO and ET systematically provide different information for height.

**Table 2:** Multi-sourced variables with high degree of discrepancy between data providers

Column	Table	DP Pair	Association
C_WarmIschemiaTime	T_Kidney_Retrieval	DSO, ET	0.009
C_BodyHeight	T_Recipient	ET, IQTIG	0.011
C_BodyWeight	T_Recipient	ET, IQTIG	0.054
C_Hypertension	T_Donor_PM	DSO, ET	0.084
C_DiuresisInterval	T_Donor_PM_Monitor	DSO, ET	0.154
C_Malign	T_Donor_PM	DSO, ET	0.383
C_Diabetes	T_Donor_PM	DSO, ET	0.388
C_Creatinine	T_Donor_PM_Chem	DSO, ET	0.561

Results of these column pairs reveal specific patterns of discrepancies between data providers. Body measurements shows particularly poor agreement: height T_Recipient.C_BodyHeight and weight T_Recipient.C_BodyWeight between ET and IQTIG suffer from inconsistent unit usage and data errors in IQTIG data.

The warm ischemia time T_Kidney_Retrieval.C_WarmIschemiaTime shows minimal association (0.01) between DSO and ET, suggesting different measurement protocols or timing definitions across data providers. Similarly, diuresis duration T_Donor_PM_Monitor.C_DiuresisInterval and creatinine values T_Donor_PM_Chem.C_Creatinine demonstrate unit inconsistencies, with DSO reporting apparently incorrect units leading to physiologically implausible measurements. These inconsistencies are plausibly attributable to historical differences in units used across Germany (SI in Eastern Germany vs conventional in Western Germany), which may create clerical errors during data entry [24].

The variables for hypertension C_Hypertension, malignancy C_Malign, and diabetes mellitus C_Diabetes in T_Donor_PM showed low inter–data-provider associations due to different categorical coding schemes employed by DSO and ET. Here in addition, the DSO only documents present status for confirmed diseases, absence is not explicitly recorded.

In summary, these findings show that for many multi-sourced variables, pairwise imputations are feasible. However, we identified important exceptions where manual inspections would be required before imputation.

### Distribution of event time data

#### Time to last follow-up

There are multiple sources for the date of last follow-up in the dataset. First, ET directly provides a date for the last follow-up in T_Transplantation.C_LastDate. In the following, we will refer to this date as the “Reported Last Follow-Up Date” (“Reported LFUD”). Furthermore, ET and IQTIG each provide a series of *0,1,…* follow-up dates in multi-sourced variable columns T_Kidney_Followup.C_Date. The corresponding combined last follow-up date can be defined as the latest date for each recipient. We call this the “Derived LFUD”. In contrast to the single ET source “Reported LFUD”, this derived date is calculated from follow-up records contributed by both ET and IQTIG in T_Kidney_Followup and therefore reflects the latest available tracked follow-up entry in that table. Consequently, “Derived LFUD” can still be earlier than “Reported LFUD” if ET reports a later follow-up date in T_Transplantation.C_LastDate that is not present in follow-up observations. Data provided by the DSO includes only donor data without recipient follow-up. recipient follow-up.

In general, IQTIG collects follow-up data during the initial hospital stay and one, two, and three years after transplantation, whereas ET may provide additional data e.g. collected in the context of a registration for re-transplantation or -allocation. For both data providers, information beyond year 3 after transplantation may therefore be incomplete, but are included in the following to provide a full picture of the data.

**Fig. 5:**
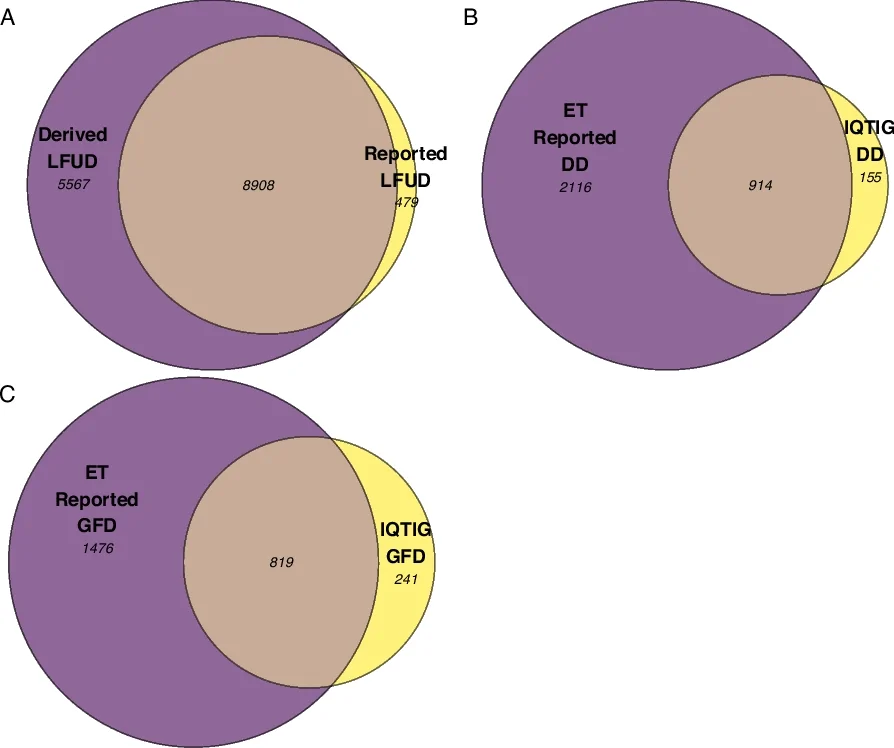
Number of recipients for whom information on the date of last follow-up (**A**), date of death (**B**) or date of graft failure (**C**) is available, separately for the different data providers that provide the information

**Fig. 6:**
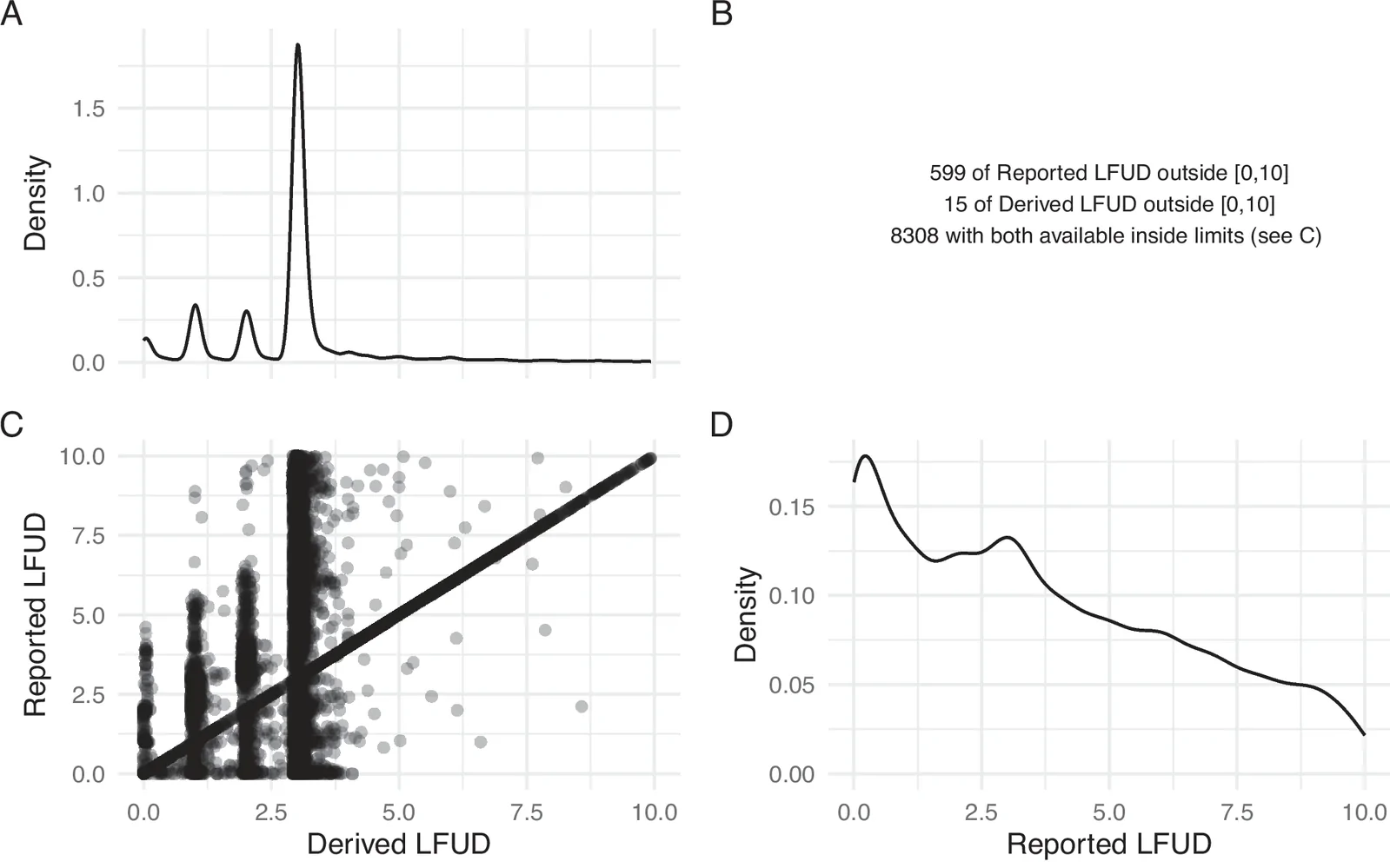
Distribution of the follow-up dates reported by different data providers. Panels **A** and **D** show density estimations of the dates, panel **B** shows date availability, and panel **C** shows a scatter plot between the dates

Recipients may either have information on the “Reported LFUD”, the “Derived LFUD” or both. This can be seen in Fig. 5. 96.8% (14,475) of the recipients have a last follow-up date according to the “Derived LFUD”. The “Reported LFUD” is given for 62.8% (9,387) of the recipients including all 3% (479) of the recipients with no available “Derived LFUD”. Figure 6 illustrates the distribution of both dates (A and D) together with their association (C).

In Fig. 6 data of 15 and 599 observations are not shown as these were lying before date of transplantation (defining timepoint 0) or more than 10 years after transplantation.

The “Derived LFUD” is clustering at 1, 2, and 3 years (A). This can be explained by the yearly follow-up schedule of patients after kidney transplantation. The “Reported LFUD” has a more even distribution of dates but also shows a peak at year 3 (D). As a consequence, the association between both dates is low (C).

#### Time to patient death

There are two sources for a death date:

- ET provides the recipient death date in T_Recipient.C_DeathDate. We will refer to this date as the “ET Reported Death Date” (“ET Reported DD”).
- In T_Kidney_Followup.C_DeathDate, a death date is reported by IQTIG if the recipient has died since the last follow-up. In the case of multiple reported dates, the earliest will be used. We will refer to this as the “IQTIG Reported Death Date” (“IQTIG Reported DD”).

The same analysis as for the last follow-up dates was done for the death dates. Figure 5 B shows that for 3,185 recipients at least one of the three dates is given and most of them are only reported by ET (2,116). The “IQTIG Reported DD” is observed for for 1,069 of all recipients.

**Fig. 7:**
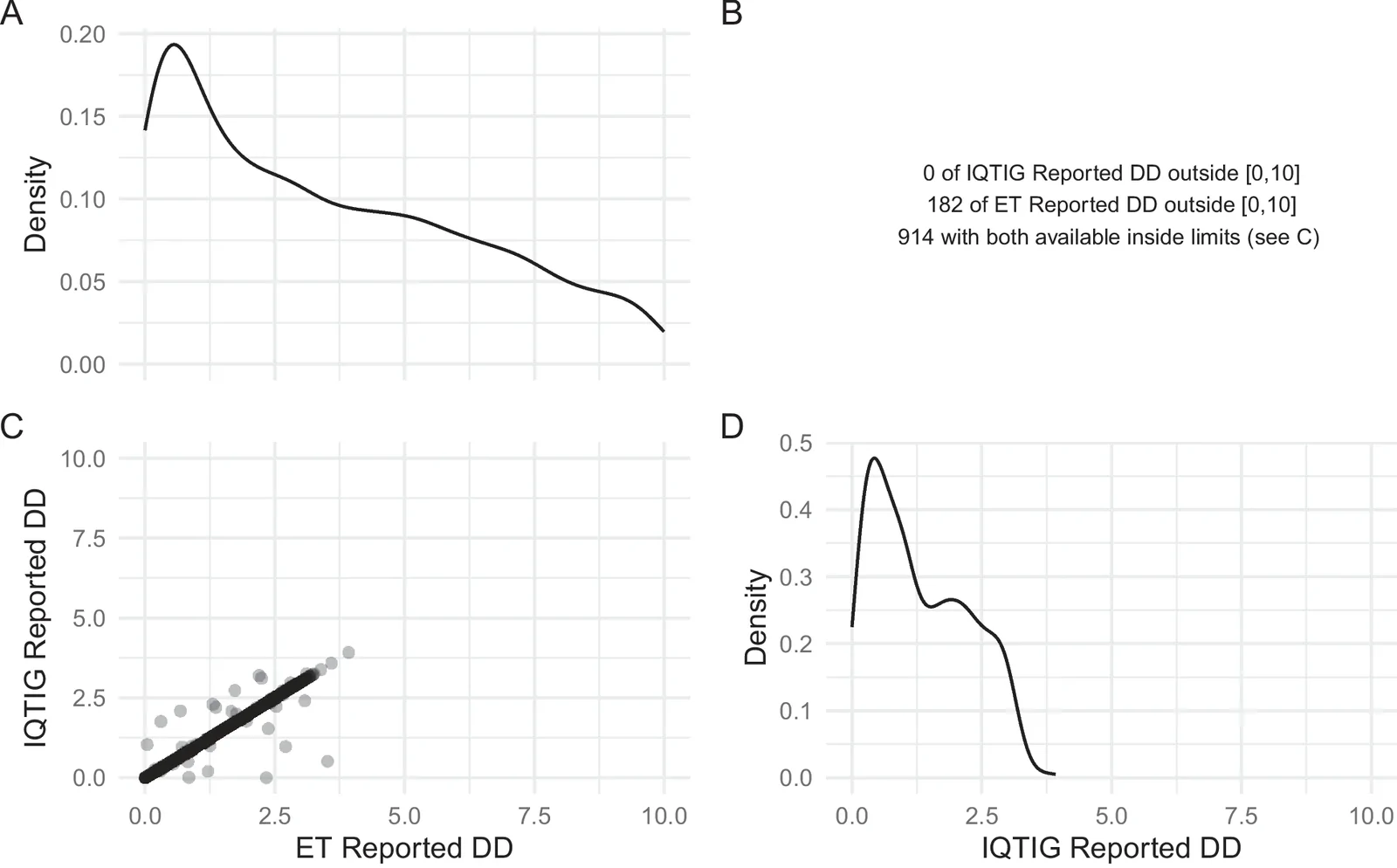
Comparison of the death dates. Panels **A** and **D** show density estimations of the dates, panel **B** shows date availability, and panel **C** shows a scatter plot between the dates

Figure 7 illustrates the distribution of the two dates (A, D) together with their associations (C).

In Fig. 7 data of 182 observations are not shown as the “ET Reported DD” was reported lying before date of transplantation (defining timepoint 0) or more than 10 years after transplantation.

We observe that some death dates are reported beyond year 3, in particular when reported by ET in “ET Reported DD”. The date sources “ET Reported DD” and “IQTIG Reported DD” agree in most cases (subfigure C).

#### Time to graft failure

Similar to recipient death, the date of graft failure is also provided in two sources.

- ET provides the date of graft failure in T_Transplantation.C_FailureDate to which we refer as “ET Reported Graft Failure Date” (“ET Reported GFD”).
- IQTIG provides a graft failure date in T_Kidney_Followup.C_FailureDate, to which we refer as “Derived Graft Failure Date” (“Derived GFD”).

The analysis of the graft failure dates results in similar observations as for the death dates. For 2,536 recipients at least one of the two dates is given as can be seen in Fig. 5B. The “IQTIG Reported GFD” is observed for 1,060 of all recipients. 1,476 of all observed graft failures are only reported in “ET Reported GFD”, while 241 are only reported by IQTIG.

**Fig. 8:**
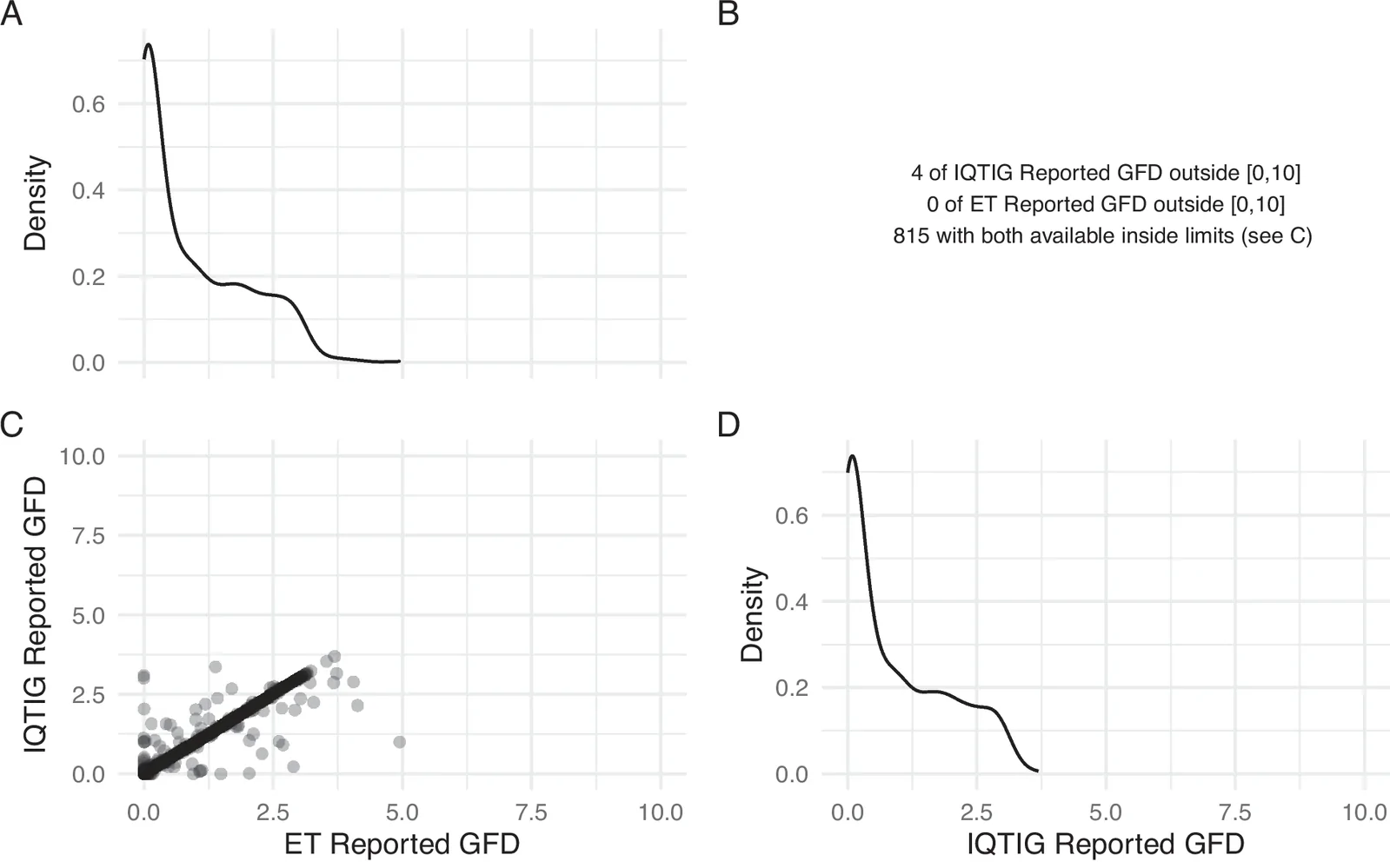
Comparison of the graft failure dates. Panels **A** and **D** show density estimations of the dates, panel **B** shows date availability, and panel **C** shows a scatter plot between the dates

Figure 8 illustrates the distribution of the two dates (A, D) together with their associations (C).

In Fig. 8 data of 4 observations are not shown as the “ET Reported DD” was repported lying before date of transplantation (defining timepoint 0) or more than 10 years after transplantation.

Results are similar to the findings for the dates of death as reported from the different data providers:. While “ET Reported GFD” and “IQTIG Reported GFD” show some agreement in many cases there is a higher number of cases, where the dates differ.

#### Consequences of different outcome definitions

To illustrate the importance of carefully defining the event time variables we estimate survival probabilities for the composite endpoint graft and patient survival when constructing the outcome from different sources.

We consider different combinations of the possible definitions for the date of death ($T^D$), the date of graft failure ($T^G$) and the date of last follow-up ($T^F$) as given in table Table 3.

**Table 3:** Comparison of the different outcome definitions

Outcome	*T*^*D*^	*T*^*G*^	*T*^*F*^	N
a) ET Reported	ET Reported DD	ET Reported GFD	Reported LFUD	10,937
b) IQTIG Reported	IQTIG Reported DD	IQTIG Reported GFD	Derived LFUD	14,471
c) Combined	Minimum of ET Reported DD and IQTIG Reported DD	Minimum of ET Reported GFD and IQTIG Reported GFD	Maximum of Reported LFUD and Derived LFUD	14,950

For each recipient *i* we define the time to composite outcome or censoring, whatever comes first as

$$\begin{aligned} T_i = \min (T^{D}_{i},T^{G}_{i},T^{F}_{i},3), \end{aligned}$$

separately for the different combinations of possible definitions of dates as given in Table 3. If $T^D_i$, $T^G_i$, and $T^F_i$ are missing, $T_i$ will be defined as missing and excluded from analyses. Otherwise the minimum is derived from the non-missing information only. The variable $\Delta _i$ indicates if $T_i$ refers to an event or a censored observation and is defined as

$$\begin{aligned} \Delta _i = \mathbb{1}\left( T_i = \min (T^D_i,T^G_i)\right) . \end{aligned}$$

By only considering dates of death and graft failure within the first 3 years after transplantation, we consider the findings indicating that information beyond year 3 may be unreliable.

For the analysis, implausible event or follow-up dates were handled using predefined plausibility rules. Recipients with at least one event or follow-up date reported more than 30 days (>1 month) before the transplantation date were excluded (4). Reported dates occurring within 30 days prior to transplantation were recoded to the transplantation date (affecting 6 dates). Dates occurring 15 years or more after transplantation were set to missing (affecting 5 dates in “Reported LFUD”).

Table 3 also gives the number of recipients for each combination. In all but one case, data are available for around 14,500 recipients. The only exception is “a) ET Reported” where both event and censoring dates are missing in around 4,000 cases.

**Fig. 9:**
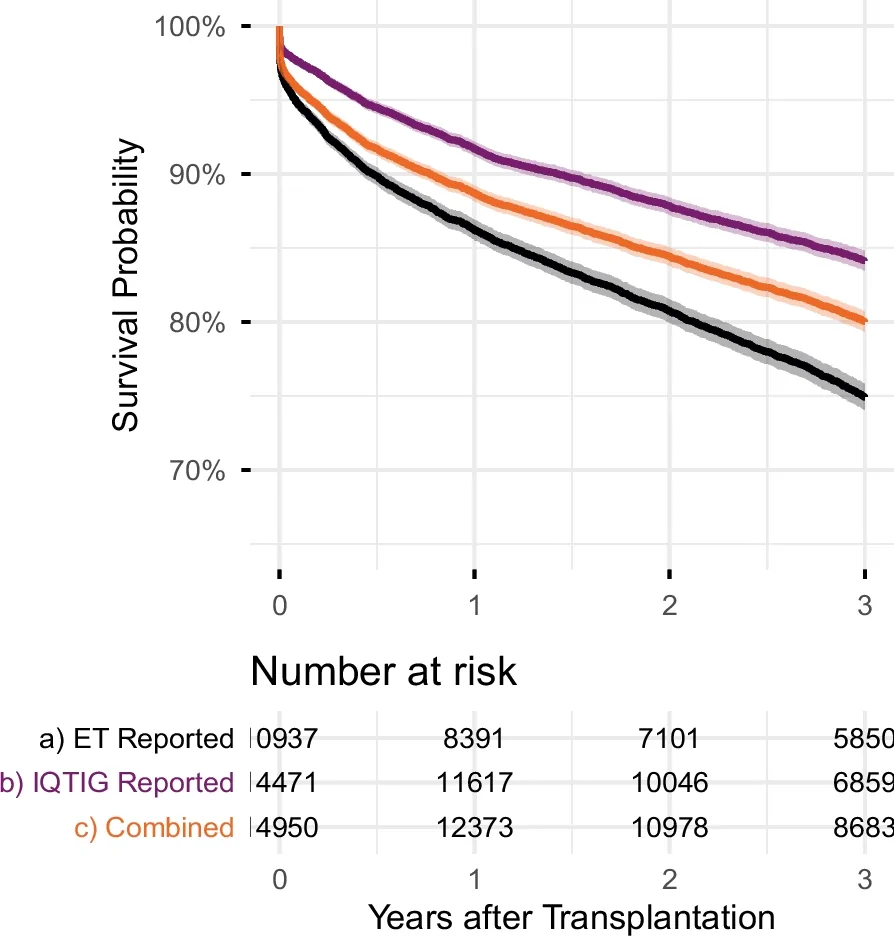
Kaplan–Meier estimates of graft and patient survival (composite endpoint) over time for recipients transplanted between 2006 and 2016, with administrative censoring at three years post-transplantation

Kaplan-Meier estimates $\hat{S}$ for the composite endpoint of graft and patient survival are calculated separately for each of the three outcome definitions. Results are shown in Fig. 9.

- When relying on information provided by ET only (“a) ET Reported”), smaller survival probabilities are estimated than with all other outcomes. This can be explained by the fact that ET reports the largest number of patient deaths and graft failures, while about 4,000 recipients without follow-up information are excluded. These likely refer mainly to survivors.
- Conversely, when relying mainly on information provided by IQTIG (“b) IQTIG Reported”), higher survival probabilities are estimated compared to the results using information from ET. This can be explained by the fact that many patient deaths and graft failures are not reported by IQTIG.
- The outcome “c) Combined” uses all information irrespective of the data provider that provided that information. As a consequence, the survival estimates lie between the estimates that rely on a single data provider only.

In summary, we therefore recommend using the “c) Combined” outcome definition for event time analyses of single or composite events.

## Discussion

Based on the finding of our initial data analysis, we propose the following recommendations for data preprocessing and analysis:

1. Tables containing a mix of cross-sectional and longitudinal data should be split by observation type prior to any imputation.
2. Flux analysis can be used to preselect variables for imputation, thereby helping to prioritize and focus efforts.
3. Multi-sourced variables should be used for imputing missing data. For important exceptions as indicated in this paper, we recommend to first harmonize units and codings before imputation using plausible value ranges and mappings.
4. Data from Eurotransplant (ET) and the German Organ Transplantation Foundation (DSO) should be prioritized for primary analyses, while data from the Institute for Quality Assurance and Transparency in Health Care (IQTIG) may serve as a supplementary source for imputation. This is in line with the role of IQTIG as a quality assurance institution.
5. When defining outcomes for time-to-event analysis, we recommend using a combined outcome that integrates information from all three data providers. Relying solely on data from a single source may lead to biased results.

Other studies using the TxReg data have employed different strategies for extracting the relevant information. von Samson-Himmelstjerna et al. [25] analyzed patient time on the waiting list for a first kidney transplantation in the “legacy data” between 2006 and 2016. While they reported using data from ET and DSO, they did not mention the use of IQTIG data. Their study population included 24,150 recipients, which is larger compared to our population of 14,954 recipients. This is probably due to different inclusion criteria. Kolbrink et al. [9] combined both “legacy data” and “new data” to analyze the impact of the 65-year age criterion for the “ET Senior Program” (ESP) versus the standard ET Kidney Allocation System (ETKAS) on time spent on the kidney transplant waiting list. They also did not reference the use of IQTIG data. Otto et al. [3] focused on data quality in the “legacy data”, also highlighting challenges related to multi-sourced variables and emphasizing that the different data providers collect data at different points in the transplantation and follow-up process. Their analysis, which covered all organ types, noted that not all recipients, donors, and transplantations are represented consistently across all tables. For our study, detailed methodology documentation for each source database (ET, DSO, IQTIG) was only partially available. We therefore relied on the TxReg handbook and publicly available registry documentation [12]. The limited transparency of source-specific documentation is itself an important limitation of the registry and is consistent with the challenges reported by Otto et al. [3].

In our study, we addressed this issue by limiting the target population to recipients with consistently documented transplantations, donors, and follow-ups across all relevant sources (see Supplementary Figures 2 to 4, Additional File 1). Before IQTIG was responsible for collecting the quality assurance data, the BQS Institute had similar role. In studies using DSO data from 2006 and 2008 - linked to BQS data - challenges were reported similar to those we observed in the registry [24, 26, 27].

Coemans et al. [28] reported a one-year death-censored graft survival of 92.0% for patients transplanted between 2006 and 2015. Using our combined outcome variable (“c) Combined”), we obtained an estimated one-year survival of 88.68% for one year and a three-year survival of 80.03%. For Iranian kidney transplant recipients, Ghelichi-Ghojogh et al. [29] reported one year survival estimates of 92.48% for graft survival and 91.27% for patient survival. For three year survival, the reported estimates were 85.08% and 86.46%.

Mortality ascertainment data in the TxReg is based on routine documentation at transplant centers (and/or aftercare providers) and subsequent reporting to the respective data providers (ET and IQTIG) [12]. This is different from for example the SRTR database in the US, which is supplemented by the Social Security Death Master File (SSDMF) [30]. The usage of relative dates in the TxReg instead of absolute dates, limits the potential analysis to external events like the COVID-19 pandemic. this also prevents robust calendar-time stratification into transplantation eras (e.g., 5-year cohorts). As a result, together with the short follow-up, analyses cannot be directly aligned to contemporary practice periods, which limits immediate clinical interpretability.

For clinicians and patients, the central question is what the TxReg can and cannot reliably support in practice.

Strengths of the TxReg data include: (1) broad national coverage across key steps of kidney transplantation care, (2) complementary information from ET, DSO, and IQTIG that can improve endpoint ascertainment when sources are combined, and (3) the possibility to reconstruct recipient-, donor-, and transplantation-level trajectories when records are consistently linked.

Important limitations include: (1) insufficient documentation of data collection processes and individual variable definitions across providers, which hampers transparent interpretation, (2) inconsistent representation of recipients, donors, and transplantations across and within tables, requiring strict inclusion criteria to ensure comparable data, and (3) missing reliable long-term follow-up data.

Accordingly, this IDA supports three robust practical conclusions: first, TxReg data can be used for clinically relevant kidney-transplant analyses when cohorts are restricted to consistently linked recipient, donor, and transplantation records; second, endpoint ascertainment is more complete when information from ET, DSO, and IQTIG is combined rather than relying on a single source; third, reproducible inference requires explicit preprocessing decisions, including source prioritization, harmonized coding/units, and transparent handling of missingness before model-based analyses.

Beyond these registry-level constraints, our study has additional limitations specific to its scope: we focused on kidney transplantation in the “legacy data” and did not include other organs or the structurally different “new data” (post-2016). Some challenges, including approximate record linkage with repeat transplantations, were outside the present scope. Kaplan-Meier analyses were used illustratively; for formal time-to-event inference in this setting, competing-risk methods are more appropriate, as emphasized by Coemans et al. [31].

## Conclusions

We demonstrated that using the TxReg data for research requires careful consideration for data-pre-processing, handling missing values and defining long-term outcomes. The reason is a complex data structure caused by different data providers providing both distinct as well as overlapping information. We identified 168 multi-sourced variables reported by multiple providers in parallel, which introduce discrepancies in coding and units but also provide opportunities for missing data imputation. Furthermore, missing data patterns are often predictable from observation types which can guide imputation strategies in future analyses.

Our initial data analysis will assist researchers in both understanding and making appropriate use of the TxReg data. In practical terms, our analyses support three actionable conclusions: (1) kidney-transplant analyses are feasible with consistently linked recipient, donor, and transplantation records, but require careful preprocessing to account for missing data and data inconsistencies, (2) combined ET/DSO/IQTIG definitions improve ascertainment compared to single-source definitions, and (3) future analyses using TxReg data should be preceded by a targeted initial data analysis to ensure appropriate handling of the registry’s structural complexities.

## Supplementary Information

Additional file 1: Supplementary Material. This file contains supplementary figures and tables referenced in the main text.

## Data Availability

The data analysis presented in this manuscript uses retrospective and anonymized data of the German Transplant Registry. These data have been supplied by the Transplant Registry Agency represented by the Gesundheitsforen Leipzig GmbH on the 11th of February 2022. Data can be requested for research purposes here: https://transplantations-register.de/en/research. The datasets generated and/or analyzed during the current study are not publicly available due to data protection regulations. Digital supplementary materials are available at https://github.com/Finesim97/txreg_ida_digital.

## Abbreviations

TxReg: German Transplantation Registry
ET: Eurotransplant Foundation
DSO: German Organ Transplantation Foundation
IQTIG: Institute for Quality Assurance and Transparency in Health Care
IDA: Initial Data Analysis
ESKD: End-Stage Kidney Disease
CKD: Chronic Kidney Disease
DP: Data Provider
