# Supplementary material for: Initial data analysis of the national German transplantation registry with a focus on kidney transplantation
Source: BMC Med Res Methodol. 2026 Oct 15;26:242. doi: 10.1186/s12874-026-03014-9 (PMC13749978; doi:10.1186/s12874-026-03014-9)
Supplement: Supplementary file 1 [file 12874_2026_3014_MOESM1_ESM.pdf]

Supplementary Material

Lukas Klein   Gunter Grieser   Carl-Ludwig Fischer-Fröhlich
Axel Rahmel   Henrik Stahl   Andreas Wienke
Antje Jahn-Eimermacher

Table of contents

1 Supplementary Table 1 1

2 Supplementary Table 2 3

3 Supplementary Information 1 and Supplementary Figure 1 6

4 Supplementary Table 3 7

5 Supplementary Table 4 17

6 Supplementary Figure 2-4 21

7 Supplementary Table 5 25

8 Supplementary Table 6-8 26

9 Supplementary Table 9 and 10 28

1 Supplementary Table 1

This table lists the original names of the tables in the export and what entities they contain.

Table 1:

Table Name in Publication	Table Name in Export	Contains Recipients	Contains Transplan-tations	Contains Donors
T_Kidney_Waitlist	warteliste_niere	Yes	No	No
T_Recip_Immuno	empfaenger_immunologie	Yes	No	No
T_Recip_Urgency	empfaenger_dringlichkeit	Yes	No	No
T_Recip_Virology	empfaenger_virologie	Yes	No	No
T_Recipient	empfaenger	Yes	No	No
T_Kidney_Med	followup_niere_medikation	No	Yes	No
T_Postop_Exam	transplantation_postop_untersuchung	No	Yes	No
T_Kidney_Followup	followup_niere	Yes	Yes	No
T_Donor_PM	spender_postmortem	No	No	Yes
T_Donor_PM_Blood_Gas	spender_postmortem_labor_blutgase	No	No	Yes
T_Donor_PM_Blood_Type	spender_postmortem_labor_blutgruppe	No	No	Yes
T_Donor_PM_Chem	spender_postmortem_labor_klinische_chemie	No	No	Yes
T_Donor_PM_Diag	spender_postmortem_diagnosen	No	No	Yes
T_Donor_PM_Exams	spender_postmortem_untersuchungen	No	No	Yes
T_Donor_PM_HLA	spender_postmortem_labor_hla	No	No	Yes
T_Donor_PM_Med	spender_postmortem_medikation	No	No	Yes
T_Donor_PM_Micro	spender_postmortem_labor_mikrobiologie	No	No	Yes
T_Donor_PM_Monitor	spender_postmortem_monitoring	No	No	Yes
T_Donor_PM_Path	spender_postmortem_labor_pathologie	No	No	Yes
T_Donor_PM_Tox	spender_postmortem_labor_toxikologie	No	No	Yes
T_Donor_PM_Urine	spender_postmortem_labor_urin	No	No	Yes
T_Donor_PM_Virology	spender_postmortem_labor_virologie	No	No	Yes
T_Donor_PM_Crossmatch	spender_postmortem_labor_crossmatch	Yes	No	Yes
T_Kidney_Retrieval	organ_entnahme_niere	No	Yes	Yes
T_Transplantation	transplantation	Yes	Yes	Yes

2 Supplementary Table 2

This table shows the original names of the columns in the export. For example `T_Transplantation.C_Destination` is the column `TBestimmungsortET` in `transplantation.csv`. The listed columns are relevant for the contents of this publication.

Table 2:

Table	Column Name in Export	Data Providers	Column Name in Publication
T_Donor_PM_Blood_Type	SPostmLaborBlutGrUntersuchungsart	DSO	C_DiagType
T_Donor_PM_Chem	SPostmLaborKCKreatininWert	DSO, ET	C_Creatinine
T_Donor_PM_Crossmatch	SPostmLaborCrossmTXCET	DSO	C_TXC
T_Donor_PM_Crossmatch	SPostmLaborCrossmXMERG	DSO	C_XMERG
T_Donor_PM_Diag	SPostmDiagnosenKlassifikation	DSO	C_DiagClass
T_Donor_PM_Diag	SPostmDiagnosenDiagnoseBeschreibung	DSO	C_DiagDescr
T_Donor_PM_Med	SPostmMedienDosisEinheit	DSO, ET	C_DosUnit
T_Donor_PM_Monitor	SPostmMonitHstillstandDauerWert	DSO, ET	C_ArrestDur
T_Donor_PM_Monitor	SPostmMonitDiureseZeitintervallWert	DSO, ET	C_DiuresisInterval
T_Donor_PM	SPostmBasisAlter	DSO, IQTIG	C_Age
T_Donor_PM	SPostmBasisGeburtsdatum	ET	C_BirthDate
T_Donor_PM	SPostmAnamnVorerkrankungenDiabetesMellitus	DSO, ET	C_Diabetes
T_Donor_PM	SPostmAnamnVorerkrankungenHypertonie	DSO, ET	C_Hypertension
T_Donor_PM	SPostmAnamnMalignitaet	DSO, ET	C_Malign
T_Donor_PM	SPostmBasisGeschlecht	DSO, ET, IQTIG	C_Sex
T_Kidney_Followup	FNDate	ET, IQTIG	C_Date
T_Kidney_Followup	FNTodesdatum	IQTIG	C_DeathDate
T_Kidney_Followup	FNPatientVerstorben	IQTIG	C_DeathIndicator
T_Kidney_Followup	FNGraftversagenDate	IQTIG	C_FailureDate
T_Kidney_Followup	FNGraftversagen	IQTIG	C_FailureIndicator
T_Kidney_Followup	FNCenter	ET	C_FolUpCenter
T_Kidney_Followup	FNFollowUpArt	ET	C_FolUpType
T_Kidney_Retrieval	ONIschaemiezeitWarmErsteWert	DSO, ET	C_WarmIschemiaTime
T_Kidney_Waitlist	WNGrunderkrankungBeschreibung	ET	C_BaseDiseaseDescr

T_Kidney_Waitlist	WNGrunderkrankung	ET, IQTIG	C_BaseDisease
T_Kidney_Waitlist	WNTransplantationenNAnzahl	ET	C_KidneyTXCount
T_Recip_Immuno	EImmDiagnostikZentrum	ET	C_DiagCenter
T_Recip_Immuno	EImmErgebnisTyp	ET	C_ResultType
T_Recip_Urgency	EDringlCode	ET	C_UrgeCode
T_Recip_Urgency	EDringlDate	ET	C_UrgencyDate
T_Recipient	EBasisGeburtsdatum	ET, IQTIG	C_BirthDate
T_Recipient	EBasisGroesseWert	ET, IQTIG	C_BodyHeight
T_Recipient	EBasisGewichtEinheit	ET, IQTIG	C_BodyWeightUnit
T_Recipient	EBasisGewichtWert	ET, IQTIG	C_BodyWeight
T_Recipient	EBasisLand	ET	C_Country
T_Recipient	EBasisTodesdatum	ET	C_DeathDate
T_Recipient	EBasisGeschlecht	ET, IQTIG	C_Sex
T_Transplantation	TVergabeProgram	ET	C_AllocationProgram
T_Transplantation	TBestimmungsort	ET	C_Destination
T_Transplantation	TPostOPOrganversagenDate	ET	C_FailureDate
T_Transplantation	TFollowUpZentrum	ET	C_FolUpCenter
T_Transplantation	TFollowUpLetztesDate	ET	C_LastDate
T_Transplantation	TOrgan	ET	C_Organ
T_Transplantation	TTransplantationszentrumRegistrierung	ET	C_RegCenter
T_Transplantation	TTransplantationszentrum	ET	C_TXCenter
T_Transplantation	TTxDate	ET, IQTIG	C_TXDate

3 Supplementary Information 1 and Supplementary Figure 1

To construct our target population, we first joined the 78,666 recipients in `T_Recipient` table and transplantations reported by ET in `T_Transplantation` on the ET recipient identifier. This lead to 44629 recipients. Afer calculating the age at the time of the transplantation by calculating the difference between `T_Recipient.C_BirthDate` and `T_Transplantation.C_TXDate`, we filtered for recipients older then 18 years and who had both columns not missing. This lead to 41,984 recipients. To only keep kidney recipients, `T_Transplantation.C_Organ` had to be either "Left Kidney" or "Right Kidney". This lead to 24,760 recipients. To filter for first-time kdiney-only recipients, we filtered recipients who had multiple observations reported by ET in `T_Transplantation`, which reduced the number of recipients to 24,054. Furthmore to filter recipients, who might have had kidney trasnplantations not listed in the TxReg data, the column `T_Kidney_Waitlist.C_KidneyTXCount` had to have a non-missing value equal or below 1. This lead to 21,283 recipients. Finally, to only include data from deceased donors, the donors for the transplantations had to have an entry in `T_Donor_PM`, leading to the final number of recipients 14,954.

The flowchart of this inclusion process is shown in Figure 1.

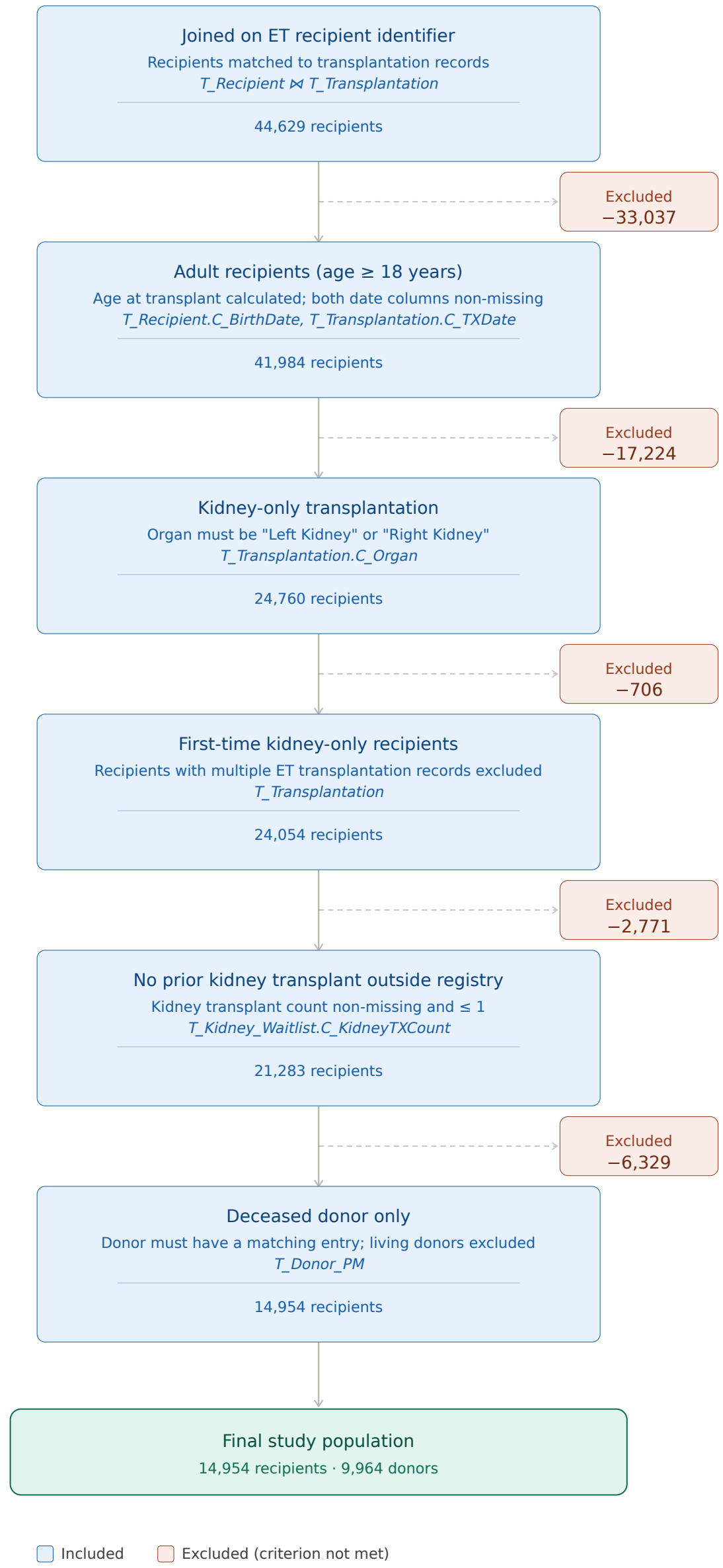

Figure 1: Flowchart of the filtering process.

4 Supplementary Table 3

This table shows the columns which were in clusters 3 and 4 of the flux analysis.

Table 3:

Cluster	Table	Column Prefix in Export	Influx	Outflux	Proportion of Missing Values
3	T_Kidney_Med	FNImmunsuppression-DateET	0.000	1.000	0.00%
3	T_Kidney_Med	FNImmunsuppression-IdTransplantations-NrET	0.000	1.000	0.00%
3	T_Kidney_Med	FNImmunsuppression-NameET	0.000	1.000	0.00%
3	T_Recip_Urgency	EDringlCodeET	0.000	1.000	0.00%
3	T_Recip_Urgency	EDringlDateET	0.000	1.000	0.00%
3	T_Recip_Urgency	EDringlId-EmpfaengerNrETET	0.000	1.000	0.00%
3	T_Recip_Urgency	EDringlOrganET	0.000	1.000	0.00%
3	T_Postop_Exam	TPostOPUntersuchung-DateET	0.000	1.000	0.00%
3	T_Postop_Exam	TPost-OPUntersuchungId-Transplantationsnummer-ETET	0.000	1.000	0.00%
3	T_Postop_Exam	TPostOPUntersuchung-KreatininWertET	0.001	0.819	0.10%
3	T_Recipient	EBasisBlutgrET	0.000	1.000	0.00%
3	T_Recipient	EBasisGeburtsdatum-ET	0.000	1.000	0.00%
3	T_Recipient	EBasisGeschlechtET	0.000	1.000	0.00%
3	T_Recipient	EBasisGewicht-EinheitET	0.000	1.000	0.00%
3	T_Recipient	EBasisGewichtWert-ET	0.000	1.000	0.00%
3	T_Recipient	EBasisGroesse-EinheitET	0.000	1.000	0.00%
3	T_Recipient	EBasisGroesseWert-ET	0.000	1.000	0.00%
3	T_Recipient	EBasisLandET	0.000	1.000	0.00%
3	T_Recipient	EBasisPLZET	0.000	1.000	0.00%

3	T_Recipient	EBasisRhesusfaktor-ET	0.000	1.000	0.00%
3	T_Recipient	EBasisZentrum-KontaktierungET	0.000	1.000	0.00%
3	T_Recipient	EBasisZentrum-RegistrierungET	0.000	1.000	0.00%
3	T_Recipient	EIdEmpfaengerNr-ETET	0.000	1.000	0.00%
3	T_Donor_PM_Crossmatch	SPostmLaborCrossm-AuftragDateDSO	0.000	1.000	0.00%
3	T_Donor_PM_Crossmatch	SPostmLaborCrossm-BefundDateDSO	0.000	1.000	0.00%
3	T_Donor_PM_Crossmatch	SPostmLabor-CrossmEmpfaenger-IDpotentiellDSO	0.000	1.000	0.00%
3	T_Donor_PM_Crossmatch	SPostmLaborCrossm-ErfahrenAmDateDSO	0.000	1.000	0.00%
3	T_Donor_PM_Crossmatch	SPostmLaborCrossm-IdDSOKennnummerDSO	0.000	1.000	0.00%
3	T_Donor_PM_Crossmatch	SPostmLaborCrossm-IdEmpfaengerNr-ETDSO	0.000	1.000	0.00%
3	T_Donor_PM_Crossmatch	SPostmLaborCrossm-IdSpenderNrETDSO	0.000	1.000	0.00%
3	T_Donor_PM_Crossmatch	SPostmLaborCrossm-LNRDSO	0.000	1.000	0.00%
3	T_Donor_PM_Crossmatch	SPostmLaborCrossm-NRDSO	0.000	1.000	0.00%
3	T_Donor_PM_Crossmatch	SPostmLaborCrossm-RangDSO	0.000	1.000	0.00%
3	T_Donor_PM_Crossmatch	SPostmLaborCrossm-TXCETDSO	0.000	1.000	0.00%
3	T_Donor_PM_Crossmatch	SPostmLaborCrossm-XMERGDSO	0.000	1.000	0.00%
3	T_Donor_PM_Crossmatch	SPostmLaborCrossm-XMESPDSO	0.077	0.907	8.40%
3	T_Donor_PM_Crossmatch	SPostmLaborCrossm-XMIGMDSO	0.056	0.933	6.00%
3	T_Donor_PM_Crossmatch	SPostmLaborCrossm-XMREGDSO	0.000	1.000	0.00%

3	T_Donor_PM_Crossmatch	SPostmLaborCrossm-XMSDateDSO	0.079	0.883	9.40%
3	T_Donor_PM_Blood_Gas	SPostmLabor-BGErfahrenAmDate-DSO	0.002	0.823	8.20%
3	T_Donor_PM_Blood_Gas	SPostmLaborBGId-DSOKennnummerDSO	0.000	0.825	8.00%
3	T_Donor_PM_Blood_Gas	SPostmLaborBGId-SpenderNrETDSO	0.000	0.825	8.00%
3	T_Donor_PM_Blood_Gas	SPostmLaborBGId-SpenderNrETET	0.000	0.975	1.00%
3	T_Donor_PM_Blood_Gas	SPostmLaborBGProbe-DateDSO	0.000	0.825	8.00%
3	T_Donor_PM_Blood_Gas	SPostmLaborBGProbe-DateET	0.000	0.975	1.00%
3	T_Donor_PM_Monitor	SPostmMonit-Blutdruck-DiastolischEinheit-ET	0.046	0.859	10.50%
3	T_Donor_PM_Monitor	SPostmMonit-Blutdruck-DiastolischWertET	0.046	0.859	10.50%
3	T_Donor_PM_Monitor	SPostmMonit-BlutdruckSystolisch-EinheitET	0.044	0.863	10.20%
3	T_Donor_PM_Monitor	SPostmMonit-BlutdruckSystolisch-WertET	0.044	0.863	10.20%
3	T_Donor_PM_Monitor	SPostmMonitDateET	0.000	0.983	1.10%
3	T_Donor_PM_Monitor	SPostmMonit-HfrequenzEinheitET	0.056	0.842	11.90%
3	T_Donor_PM_Monitor	SPostmMonit-HfrequenzWertET	0.056	0.842	11.90%
3	T_Donor_PM_Monitor	SPostmMonitId-SpenderNrETET	0.000	0.983	1.10%
3	T_Donor_PM_Monitor	SPostmMonit-TemperaturEinheit-ET	0.074	0.816	14.20%
3	T_Donor_PM_Monitor	SPostmMonit-TemperaturWertET	0.074	0.816	14.20%

3	T_Donor_PM_Urine	SPostmLabor-URIErfahrenAmDate-DSO	0.002	0.876	4.90%
3	T_Donor_PM_Urine	SPostmLabor-URIGlukoseDSO	0.015	0.851	6.70%
3	T_Donor_PM_Urine	SPostmLaborURIId-DSOKennnummerDSO	0.000	0.880	4.60%
3	T_Donor_PM_Urine	SPostmLaborURIId-SpenderNrETDSO	0.000	0.880	4.60%
3	T_Donor_PM_Urine	SPostmLabor-URIProbeDateDSO	0.000	0.880	4.60%
3	T_Donor_PM_Urine	SPostmLabor-URIProteinDSO	0.011	0.858	6.10%
3	T_Donor_PM_Virology	SPostmLabor-VIRErfahrenAmDate-DSO	0.001	0.796	12.30%
3	T_Donor_PM_Virology	SPostmLaborVIRId-DSOKennnummerDSO	0.000	0.800	12.00%
3	T_Donor_PM_Virology	SPostmLaborVIRId-SpenderNrETDSO	0.000	0.800	12.00%
3	T_Donor_PM_Virology	SPostmLaborVIRId-SpenderNrETET	0.000	0.823	8.00%
3	T_Donor_PM_Virology	SPostmLabor-VIRProbeDateDSO	0.000	0.800	12.00%
3	T_Donor_PM_Virology	SPostmLabor-VIRProbeDateET	0.000	0.823	8.00%
3	T_Donor_PM	SPostmAnamn-AlkoholkonsumDSO	0.009	0.756	12.60%
3	T_Donor_PM	SPostmAnamn-DrogenkonsumDSO	0.016	0.749	13.20%
3	T_Donor_PM	SPostmAnamnRaucher-DSO	0.009	0.755	12.60%
3	T_Donor_PM	SPostmAnamnRaucher-ET	0.014	0.828	8.10%
3	T_Donor_PM	SPostmBasis-Abschaltung-LebenserhaltungDate-ET	0.000	0.842	6.70%
3	T_Donor_PM	SPostmBasisAlter-DSO	0.000	0.774	11.20%

3	T_Donor_PM	SPostmBasis-AufnahmeDateET	0.000	0.842	6.70%
3	T_Donor_PM	SPostmBasis-BeatmungBeginnDate-ET	0.001	0.842	6.80%
3	T_Donor_PM	SPostmBasisBlutgr-ET	0.000	0.842	6.70%
3	T_Donor_PM	SPostmBasis-DSORegionDSO	0.000	0.774	11.20%
3	T_Donor_PM	SPostmBasis-ExplantationDateET	0.002	0.841	6.90%
3	T_Donor_PM	SPostmBasis-GeburtsdatumET	0.000	0.842	6.70%
3	T_Donor_PM	SPostmBasis-GeschlechtDSO	0.000	0.774	11.20%
3	T_Donor_PM	SPostmBasis-GeschlechtET	0.000	0.842	6.70%
3	T_Donor_PM	SPostmBasisGewicht-EinheitDSO	0.000	0.774	11.20%
3	T_Donor_PM	SPostmBasisGewicht-EinheitET	0.000	0.842	6.70%
3	T_Donor_PM	SPostmBasisGewicht-WertDSO	0.000	0.774	11.20%
3	T_Donor_PM	SPostmBasisGewicht-WertET	0.000	0.842	6.70%
3	T_Donor_PM	SPostmBasisGroesse-EinheitDSO	0.000	0.774	11.20%
3	T_Donor_PM	SPostmBasisGroesse-EinheitET	0.000	0.842	6.70%
3	T_Donor_PM	SPostmBasisGroesse-WertDSO	0.000	0.774	11.20%
3	T_Donor_PM	SPostmBasisGroesse-WertET	0.000	0.842	6.70%
3	T_Donor_PM	SPostmBasis-HstillstandDauer-EinheitET	0.000	0.842	6.70%
3	T_Donor_PM	SPostmBasis-HstillstandDauer-WertET	0.000	0.842	6.70%

3	T_Donor_PM	SPostmBasis-HypotensiveEpisode-DauerEinheitET	0.000	0.842	6.70%
3	T_Donor_PM	SPostmBasis-HypotensiveEpisode-DauerWertET	0.000	0.842	6.70%
3	T_Donor_PM	SPostmBasis-Intensivbehandlung-BeginnDateET	0.001	0.842	6.80%
3	T_Donor_PM	SPostmBasisMeldung-KHDSO	0.000	0.774	11.20%
3	T_Donor_PM	SPostmBasisMeldung-KHOrtDSO	0.000	0.774	11.20%
3	T_Donor_PM	SPostmBasisMeldung-ZeitpunktDSO	0.000	0.774	11.20%
3	T_Donor_PM	SPostmBasisPostm-TypET	0.000	0.842	6.70%
3	T_Donor_PM	SPostmBasis-Registrierungszeitpunkt-ETDSO	0.007	0.763	12.10%
3	T_Donor_PM	SPostmBasis-RhesusfaktorET	0.000	0.842	6.70%
3	T_Donor_PM	SPostmBasisSpender-AusAuslandDSO	0.000	0.774	11.20%
3	T_Donor_PM	SPostmBasis-SpenderartET	0.000	0.842	6.70%
3	T_Donor_PM	SPostmBasis-SpenderstatusDSO	0.000	0.774	11.20%
3	T_Donor_PM	SPostmBasisStatus-DarmDSO	0.003	0.770	11.60%
3	T_Donor_PM	SPostmBasisStatus-LesplitLinksDSO	0.019	0.756	13.00%
3	T_Donor_PM	SPostmBasisStatus-LesplitRechtsDSO	0.019	0.757	13.00%
3	T_Donor_PM	SPostmBasis-UrinkatheterBeginn-DateET	0.009	0.835	7.60%
3	T_Donor_PM	SPostmBasis-ZeitpunktHirntodET	0.000	0.842	6.70%
3	T_Donor_PM	SPostmId-DSOKennnummerDSO	0.000	0.774	11.20%

3	T_Donor_PM	SPostmIdSpenderNr-ETDSO	0.000	0.774	11.20%
3	T_Donor_PM	SPostmIdSpenderNr-ETET	0.000	0.842	6.70%
3	T_Donor_PM	SPostmRechtl-EignungEntscheidung-DarmDSO	0.000	0.774	11.20%
3	T_Donor_PM	SPostmRechtl-EignungEntscheidung-HDSO	0.000	0.774	11.20%
3	T_Donor_PM	SPostmRechtl-EignungEntscheidung-LeDSO	0.000	0.774	11.20%
3	T_Donor_PM	SPostmRechtl-EignungEntscheidung-LuLinksDSO	0.000	0.774	11.20%
3	T_Donor_PM	SPostmRechtl-EignungEntscheidung-LuRechtsDSO	0.000	0.774	11.20%
3	T_Donor_PM	SPostmRechtl-EignungEntscheidung-NLinksDSO	0.000	0.774	11.20%
3	T_Donor_PM	SPostmRechtl-EignungEntscheidung-NRechtsDSO	0.000	0.774	11.20%
3	T_Donor_PM	SPostmRechtl-EignungEntscheidung-PDSO	0.000	0.774	11.20%
3	T_Donor_PM	SPostmRechtl-Eignung-Entscheidungszeitpunkt-DSO	0.000	0.774	11.20%
3	T_Kidney_Retrieval	ONAnzahlArterien-DSO	0.000	0.912	4.30%
3	T_Kidney_Retrieval	ONAnzahlArterienET	0.001	0.974	0.40%
3	T_Kidney_Retrieval	ONAnzahlVenenDSO	0.000	0.912	4.30%
3	T_Kidney_Retrieval	ONAnzahlVenenET	0.001	0.973	0.40%
3	T_Kidney_Retrieval	ONArtLaengeUreter-DSO	0.002	0.910	4.50%
3	T_Kidney_Retrieval	ONArteriePatchDSO	0.002	0.911	4.40%
3	T_Kidney_Retrieval	ONArteriePatchET	0.003	0.969	0.60%

3	T_Kidney_Retrieval	ONEnBlocEntnahme-DSO	0.029	0.880	7.30%
3	T_Kidney_Retrieval	ONEntnahmeDateDSO	0.000	0.914	4.20%
3	T_Kidney_Retrieval	ONEntnahmekrankenhaus-ET	0.001	0.975	0.40%
3	T_Kidney_Retrieval	ONEntnahmezentrum-DSO	0.000	0.914	4.20%
3	T_Kidney_Retrieval	ONHeparinDateET	0.018	0.941	2.30%
3	T_Kidney_Retrieval	ONHeparinVolumen-EinheitET	0.000	0.982	0.20%
3	T_Kidney_Retrieval	ONHeparinVolumen-WertET	0.018	0.941	2.30%
3	T_Kidney_Retrieval	ONIdDSOKennnummer-DSO	0.000	0.914	4.20%
3	T_Kidney_Retrieval	ONIdSpenderNr-ETDSO	0.000	0.914	4.20%
3	T_Kidney_Retrieval	ONIdSpenderNrETET	0.000	0.982	0.20%
3	T_Kidney_Retrieval	ONId-Transplantationsnummer-ETET	0.000	0.982	0.20%
3	T_Kidney_Retrieval	ONIschaemiezeitWarm-ErsteEinheitET	0.000	0.982	0.20%
3	T_Kidney_Retrieval	ONLaengeUreterET	0.003	0.970	0.60%
3	T_Kidney_Retrieval	ONNephrektomieDate-ET	0.001	0.976	0.30%
3	T_Kidney_Retrieval	ONOPZeiten-CrossclampDateDSO	0.000	0.914	4.20%
3	T_Kidney_Retrieval	ONOPZeitenNahtDate-DSO	0.000	0.914	4.20%
3	T_Kidney_Retrieval	ONOPZeitenSchnitt-DateDSO	0.000	0.914	4.20%
3	T_Kidney_Retrieval	ONOrganTypDSO	0.000	0.914	4.20%
3	T_Kidney_Retrieval	ONOrganTypET	0.000	0.982	0.20%
3	T_Kidney_Retrieval	ONOrganqualitaetDSO	0.004	0.908	4.70%
3	T_Kidney_Retrieval	ONOrganqualitaetET	0.008	0.961	1.20%
3	T_Kidney_Retrieval	ONPerfusionKalt-StartDateET	0.000	0.982	0.20%
3	T_Kidney_Retrieval	ONPerfusionLoesung-ET	0.001	0.974	0.40%
3	T_Kidney_Retrieval	ONPerfusion-MaschinellDSO	0.034	0.871	8.00%

3	T_Kidney_Retrieval	ONPerfusion-MaschinellET	0.083	0.858	8.90%
3	T_Kidney_Retrieval	ONPerfusion-QualitaetDSO	0.002	0.911	4.40%
3	T_Kidney_Retrieval	ONPerfusion-QualitaetET	0.004	0.967	0.70%
3	T_Kidney_Retrieval	ONPerfusionVolumen-EinheitET	0.000	0.982	0.20%
3	T_Kidney_Retrieval	ONPerfusionVolumen-WertET	0.003	0.971	0.50%
3	T_Kidney_Retrieval	ONVenenPatchDSO	0.005	0.909	4.70%
3	T_Kidney_Retrieval	ONVenenPatchET	0.007	0.962	1.00%
3	T_Donor_PM_Tox	SPostmLabor-TOXErfahrenAmDate-DSO	0.073	0.846	11.80%
3	T_Donor_PM_Tox	SPostmLaborTOXId-DSOKennnummerDSO	0.000	1.000	0.00%
3	T_Donor_PM_Tox	SPostmLaborTOXId-SpenderNrETDSO	0.000	1.000	0.00%
3	T_Donor_PM_Tox	SPostmLabor-TOXProbeDateDSO	0.000	1.000	0.00%
3	T_Donor_PM_Tox	SPostmLabor-TOXProbenmaterial-DSO	0.069	0.905	8.30%
3	T_Recip_Immuno	EImmAutoantikoerper-ET	0.056	0.896	8.20%
3	T_Recip_Immuno	EImmDiagnostik-Screeningverfahren-ET	0.056	0.896	8.20%
3	T_Recip_Immuno	EImmDiagnostik-ZentrumET	0.000	1.000	0.00%
3	T_Recip_Immuno	EImmEingabeDateET	0.000	1.000	0.00%
3	T_Recip_Immuno	EImmErgebnisTypET	0.000	1.000	0.00%
3	T_Recip_Immuno	EImmIdEmpfaenger-NrETET	0.000	1.000	0.00%
3	T_Recip_Immuno	EImmPRAEinheitET	0.056	0.896	8.20%
3	T_Recip_Immuno	EImmPRAWertET	0.056	0.896	8.20%
3	T_Recip_Immuno	EImmProbeDateET	0.000	1.000	0.00%
3	T_Donor_PM_Diag	SPostmDiagnosen-Diagnose-BeschreibungDSO	0.000	0.786	14.00%

3	T_Donor_PM_Diag	SPostmDiagnosen-DiagnoseICD10DSO	0.000	0.786	14.00%
3	T_Donor_PM_Diag	SPostmDiagnosen-ErfahrenAmDateDSO	0.000	0.786	14.00%
3	T_Donor_PM_Diag	SPostmDiagnosen-ErkrankungBeginn-DateDSO	0.000	0.786	14.00%
3	T_Donor_PM_Diag	SPostmDiagnosenId-DSOKennnummerDSO	0.000	0.786	14.00%
3	T_Donor_PM_Diag	SPostmDiagnosenId-SpenderNrETDSO	0.000	0.786	14.00%
3	T_Donor_PM_Diag	SPostmDiagnosen-KlassifikationDSO	0.000	0.786	14.00%
4	T_Recipient	EBasis-BluttransfusionVor-RegistrierungET	0.332	0.567	35.60%
4	T_Recipient	EBasisNationalitaet-ET	0.488	0.499	49.10%
4	T_Donor_PM_Crossmatch	SPostmLaborCrossm-XMBZDSO	0.460	0.458	49.30%
4	T_Donor_PM_Crossmatch	SPostmLaborCrossm-XMIGGDSO	0.584	0.391	59.40%
4	T_Donor_PM_Crossmatch	SPostmLaborCrossm-XMTZDSO	0.434	0.498	46.10%
4	T_Kidney_Retrieval	ONAnzahlUreterDSO	0.373	0.540	41.50%

5 Supplementary Table 4

This table shows the results of the decision tree analysis. Only trees with any predictors are included, as postpruning might have removed all predictors.

Table 4:

Table (Data provider)	Test RMSE	Predictors
T_Kidney_Med (ET)	0.000	FNImmunsuppressionName
T_Recip_Immuno (ET)	0.029	EImmErgebnisTyp, EImmDiagnostik- Zentrum
T_Donor_PM_Diag (DSO)	0.033	SPostmDiagnosenKlassifikation, SPostmDiagnosenHirnschaedigung-Art

T_Kidney_Retrieval (ET)	0.047	ONEntnahmekrankenhaus
T_Recip_Urgency (ET)	0.055	EDringlCode, EDringlOrgan
T_Donor_PM_Crossmatch (DSO)	0.062	SPostmLaborCrossmTXCET, SPostm- LaborCrossmXMERG, SPostmLabor- CrossmXMIGM
T_Transplantation (IQTIG)	0.069	TSpendeKompatibelNP, TKomplikationIntraPost- OperationAllgmeinNP, TPost- OPFunktionsaufnahmeTransplantatN
T_Donor_PM_Med (DSO)	0.071	SPostmMedienDosisEinheit, SPostm- MedienApplikationsform
T_Donor_PM (ET)	0.076	SPostmBasisHypotensiveEpisode- DauerWert, SPostmBasisGewicht- Wert, SPostmBasisGroesse- Wert, SPostmBasisHstillstand- DauerWert, SPostmAnamn- VorerkrankungenHypertonie, SPostm- AnamnMalignitaet, SPostmBasis- Geschlecht
T_Recipient (ET)	0.078	EBasisZentrumKontaktierung, EBasisZentrumRegistrierung, EBasisGeschlecht, EBasisGroesse- Wert
T_Donor_PM (IQTIG)	0.086	SPostmBasisBeatmungDauerWert
T_Transplantation (ET)	0.089	TFollowUpZentrum, TTransplantationszentrum- Registrierung, TTransplantationszentrum, TDringl
T_Kidney_Retrieval (DSO)	0.093	ONEntnahmezentrum

T_Donor_PM_Blood_Gas (ET)	0.098	SPostmLaborBGpHO2100Prozent, SPostmLaborBGpO2O2100Prozent-Einheit, SPostmLaborBGp-O2Einheit, SPostmLabor-BGO2SaettigungO2100ProzentWert, SPostmLaborBGFiO2Wert, SPostm- LaborBGPEEPWert, SPostmLabor-BGBaseExcessO2100ProzentWert, SPostmLaborBGHCO3O2100Prozent-Wert, SPostmLaborBGO2Saettigung-Wert
T_Donor_PM_Path (DSO)	0.101	SPostmLaborPATProbenmaterial
T_Kidney_Followup (ET)	0.102	FNCenter, FNFollowUpArt, FNDialyse, FNHerkrankung-Koronar, FNHepatitisChronisch, FNDiabetesMellitus, FNHypertonie, FNKrankheitenAndere, FNTeilnahme-KlinischeStudieImmunsuppressive-Medikamente, FNInduktionstherapie, FNVerschlusskrankheitArteriell
T_Donor_PM_Monitor (ET)	0.102	SPostmMonitHstillstandDauerWert
T_Donor_PM_Blood_Type (DSO)	0.115	SPostmLaborBlutGr-Untersuchungsart
T_Kidney_Followup (IQTIG)	0.119	FNPatientVerstorben, FNGraftversagen, FNInstitutionskennzeichen
T_Donor_PM_Micro (DSO)	0.120	SPostmLaborMICERERG, SPostmLabor-MICProbenmaterial, SPostmLabor- MICKeimzahl

T_Donor_PM_HLA (DSO)	0.128	SPostmLaborHLADRDNA1, SPostm-LaborHLADRBR1ET, SPostmLabor-HLADQDNA1, SPostmLaborHLADQBR1ET, SPostmLaborHLAProbenmaterial, SPostmLaborHLADQDNA2, SPostm-LaborHLADRSP1ET, SPostmLabor-HLADRSP2ET
T_Donor_PM_Chem (DSO)	0.130	SPostmLaborKCProbenmaterial, SPostmLaborKCKalziumkonzentration-Wert, SPostmLabor-KCNatriumkonzentrationWert
T_Donor_PM_Urine (DSO)	0.164	SPostmLaborURIProtein, SPostm- LaborURIBakterien, SPostmLabor- URILeukozyten, SPostmLabor- URIGlukose
T_Donor_PM_Monitor (DSO)	0.168	SPostmMonitHstillstandDauer-Wert, SPostmMonitBlutdruck-SystolischWert, SPostmMonit-HypotensionDauerWert, SPostm-MonitBlutdruckDiastolischWert
T_Kidney_Waitlist (ET)	0.181	WNRegistrierungszentrum, WNGrunderkrankungBeschreibung, WNGrunderkrankung, WNGrunderkrankungBeschreibung-ICD10, WNGrunderkrankungICD10
T_Donor_PM_Virology (DSO)	0.185	SPostmLaborVIRHBSAK, SPostmLabor-VIRProbenmaterial, SPostmLabor- VIRHBCAK, SPostmLaborVIRCMVIg- M, SPostmLaborVIRToxoplasmose, SPostmLaborVIRHIVAg
T_Donor_PM_Virology (ET)	0.189	SPostmLaborVIRMeningitis, SPostm- LaborVIRSepsis, SPostmLabor- VIRHBsAb, SPostmLaborVIRHIVAg, SPostmLaborVIRLues, SPostmLabor- VIRHBcAb

T_Donor_PM_Tox (DSO)	0.202	SPostmLaborTOXAMS, SPostmLabor- TOXBDS, SPostmLaborTOXBTS, SPostm- LaborTOXOPS, SPostmLaborTOXABS, SPostmLaborTOXADS
T_Donor_PM_Chem (ET)	0.204	SPostmLaborKCNatriumkonzentration- Wert, SPostmLabor- KCKaliumkonzentrationWert
T_Donor_PM (DSO)	0.239	SPostmBasisDSORegion, SPostm- AnamnAlkoholkonsum, SPostmAnamn- Raucher

6 Supplementary Figure 2-4

The number of subject types (recipients, donors, and transplantations) was counted by observing the total number of unique identifiers of each type across all tables from all data providers. The availability of data for each subject type was analyzed by counting the number of unique identifiers in each table. The co-occurrence of subjects together across tables was analyzed by counting the number of subject identifiers that occur together in groups of tables.

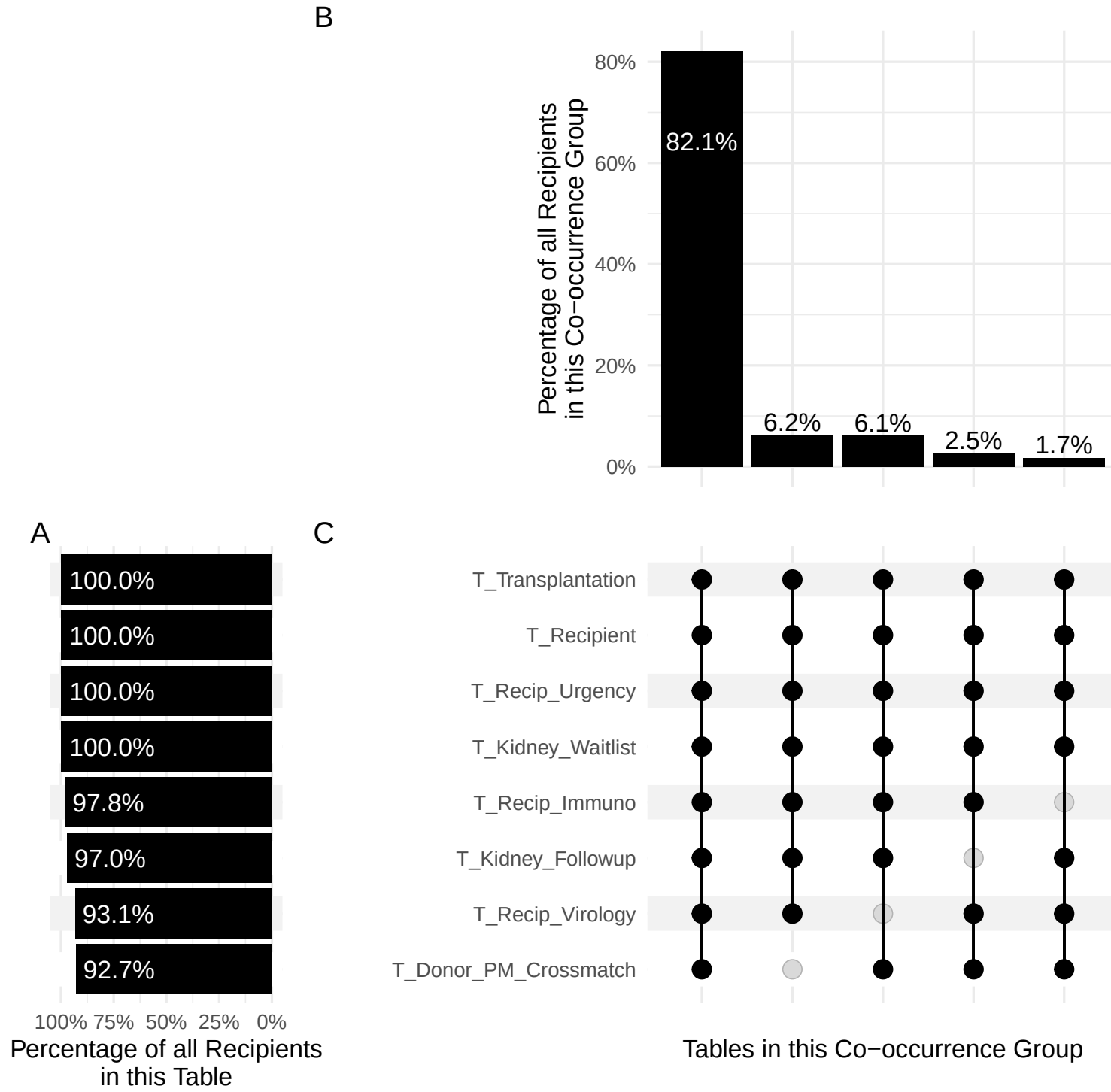

Figure 2: Recipient data availability and co-occurrence: In A the fraction of recipients with data in each table is shown. In B and C co-occurring groups of recipients are visualized. The different groups are visible on the x-axis. B shows the fraction of recipients referenced in each group, while C shows the tables which provide data for these recipients. For example, all recipients have data in the table `T_Transplantation` and the largest group of recipients is has data in all tables.

Data for 14,954 recipients are collected in 8 tables. To understand how much data is available for each recipient, we will look at the fraction of all recipients who have any data in each table. In Figure 2 A this availability is shown for each table with recipient data. All tables provide at least some data for 13,866 (93%) of all recipients. `T_Recipient`, `T_Recip_Urgency`, `T_Transplantation` and `T_Kidney_Waitlist` contain data for all recipients. However, data in `T_Recipient`, `T_Kidney_Waitlist` and `T_Transplantation` was necessary for the creation of the target population. Thus, the availability of data in these tables was a selection criterion, leading to the 100% availability. To find groups of recipients, who occur in the same tables, co-occurrence of recipients was analyzed. These groups are relevant for the analysis and modeling tasks, where conditional availability needs to be accounted for. In Figure 2 B the number of recipients in each group are shown, while in Figure 2 C the tables in which these recipients occur are shown. For most recipients (12,271 (82%)) data is available in all tables. The remaining recipients have data available all tables except one.

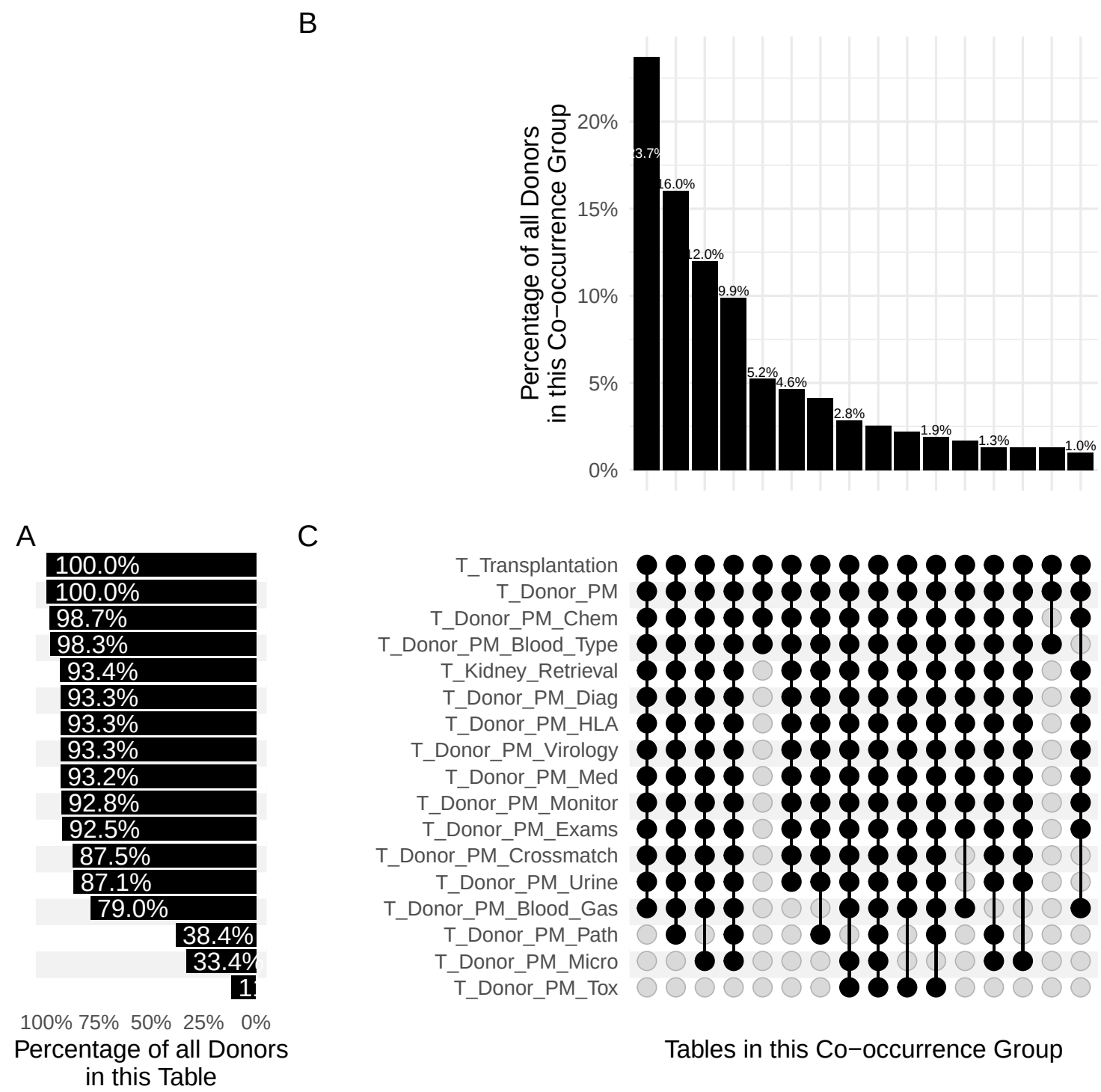

Figure 3: Donor data availability and co-occurrence: In A the fraction of donors with data in each table is shown. In B and C co-occuring groups of donors are visualized. The different groups are visible on the x-axis. B shows the fraction of donors referenced in each group, while C shows the tables which provide data for these donors. For example, all donors have data in the table `Transplantation` and the largest group of donors has data in all tables except `Donor PM Path`, `Donor PM Micro` and `Donor PM Tox`.

Data for 9,964 donors are collected in 17 tables. Data availability is shown in Figure 3 A. Of all donors 79% have at least some data in all tables except in `T_Donor_PM_Path`, `T_Donor_PM_Micro` and `T_Donor_PM_Tox`. These only contain data for 38%, 33% and 11% of all donors respectively. `T_Donor_PM` and `T_Transplantation` contain data for all donors. This is due to the fact that availability in these tables was also necessary for the creation of the target population. The co-occurrence analysis for all occurence groups containing at least 1% of all donors is shown in Figure 3 B and C. Only 3% of all donors appear in all tables. 24% of donors appear in all tables except the three mentioned above. Most other donors appear in all tables except a few. Two groups visible in Figure 3 B and C are outliers. In these groups there are 5% and 1% of all donors respectively. The first group only occurs in `T_Transplantation`, `T_Donor_PM`, `T_Donor_PM_Chem` and `T_Donor_PM_Blood_Type`. The second group appears in the same tables except `T_Donor_PM_Chem`. These might be donors for which most data was

not digitized or reported.

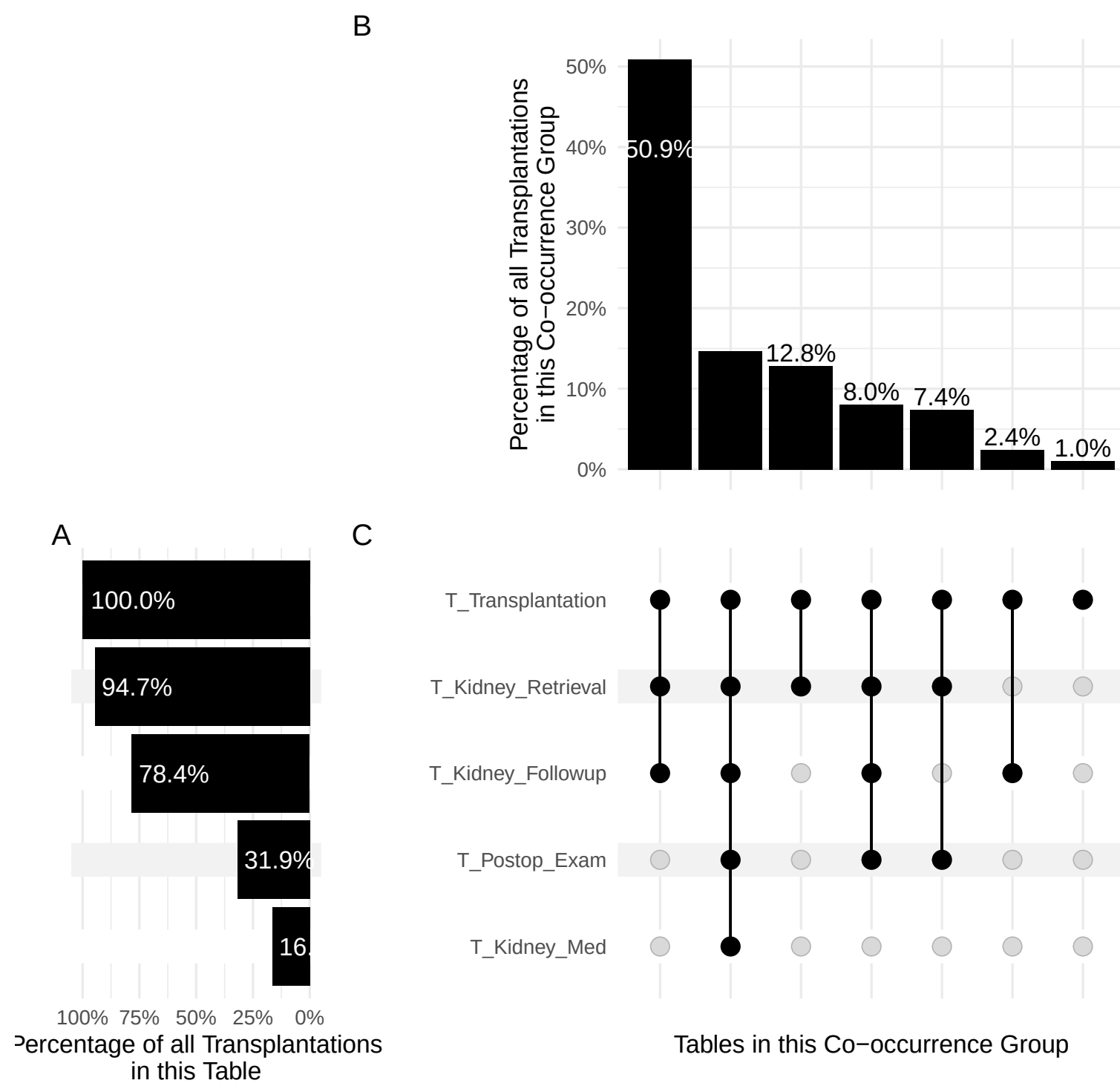

Figure 4: Transplantation data availability and co-occurrence: In A the fraction of transplantations with data in each table is shown. In B and C co-occurring groups of transplantations are visualized. The different groups are visible on the x-axis. B shows the fraction of transplantations referenced in each group, while C shows the tables which provide data for these transplantations. For example, all transplantations have data in the table `Transplantation` and the largest group of transplantations has data in the tables `Transplantation`, `Kidney Retrieval` and `Kidney Followup`.

Data for 14,954 transplantations are collected in 5 tables. Data availability is shown in Figure 4 A. Only `Transplantation` has data for all transplantations. Data in this table was again necessary for the creation of the target population. Follow-up data in `T_Kidney_Followup` is available for 78% of all transplantations. Only 32% and 17% of transplantations occur in `T_Donor_PM_Exams` and `T_Kidney_Med`. These might only have data when a post-operative examination or additional medication was necessary. Co-occurrence analysis for all groups containing at least 1% of all transplantations is shown in Figure 4 B and C. The largest group of transplantations representing 50.9% of all transplantations occurs in `T_Transplantation`, `T_Kidney_Retrieval` and `T_Kidney_Followup`. Transplantations are only referred to by ET. The other data providers use the recipient and donor together or alone to refer to the

transplantations.

7 Supplementary Table 5

The following table shows which data provider contributed to which table.

Table 5:

Table	ET	IQTIG	DSO
T_Kidney_Med	Yes	No	No
T_Postop_Exam	Yes	No	No
T_Recip_Immuno	Yes	No	No
T_Recip_Urgency	Yes	No	No
T_Recip_Virology	Yes	No	No
T_Kidney_Followup	Yes	Yes	No
T_Kidney_Waitlist	Yes	Yes	No
T_Recipient	Yes	Yes	No
T_Transplantation	Yes	Yes	No
T_Donor_PM_Crossmatch	No	No	Yes
T_Donor_PM_Tox	No	No	Yes
T_Donor_PM_Blood_Gas	Yes	No	Yes
T_Donor_PM_Diag	Yes	No	Yes
T_Donor_PM_Exams	Yes	No	Yes
T_Donor_PM_HLA	Yes	No	Yes
T_Donor_PM_Med	Yes	No	Yes
T_Donor_PM_Micro	Yes	No	Yes
T_Donor_PM_Monitor	Yes	No	Yes
T_Donor_PM_Path	Yes	No	Yes
T_Donor_PM_Urine	Yes	No	Yes
T_Donor_PM_Virology	Yes	No	Yes
T_Donor_PM_Blood_Type	No	Yes	Yes
T_Donor_PM	Yes	Yes	Yes
T_Donor_PM_Chem	Yes	Yes	Yes
T_Kidney_Retrieval	Yes	Yes	Yes

8 Supplementary Table 6-8

The following tables show whether the tables in the export contained long data or wide data. It is important to note, that there are sometimes columns which only have values for one of these observations, encoding wide data in the long format.

Table 6:

Table	Unique Rec. Count	Data provider	Avg. Rec. Observations
`T_Kidney_Waitlist`	14954	ET	1.039655
		IQTIG	1.002371
`T_Recip_Urgency`		ET	4.194262
`T_Recipient`		ET	1.000000
		IQTIG	1.000000
`T_Transplantation`		ET	1.000000
		IQTIG	1.597549
`T_Recip_Immuno`	14629	ET	13.596760
`T_Kidney_Followup`	14499	ET	1.277436
		IQTIG	2.564992
`T_Recip_Virology`	13915	ET	2.410061
`T_Donor_PM_Crossmatch`	13866	DSO	4.593033

Table 7:

Table	Unique Don. Count	Data provider	Avg. Don. Observations
`T_Donor_PM`	9964	DSO	1.000000
		ET	1.000000
		IQTIG	1.000000
`T_Transplantation`		ET	1.500803
		IQTIG	1.648732
`T_Donor_PM_Chem`	9830	DSO	3.224760
		ET	3.276774
		IQTIG	1.460669
`T_Donor_PM_Blood_Type`	9796	DSO	2.465469
		IQTIG	2.061320

T_Kidney_Retrieval	9311	DSO	1.535888
		ET	1.520245
		IQTIG	1.000000
T_Donor_PM_Diag	9300	DSO	6.449079
		ET	1.000000
T_Donor_PM_HLA	9298	DSO	1.002716
		ET	2.156264
T_Donor_PM_Virology	9297	DSO	1.386685
		ET	1.381055
T_Donor_PM_Med	9288	DSO	9.100599
		ET	4.068515
T_Donor_PM_Monitor	9249	DSO	1.736944
		ET	1.868837
T_Donor_PM_Exams	9221	DSO	4.518227
		ET	4.227865
T_Donor_PM_Crossmatch	8716	DSO	7.306907
T_Donor_PM_Urine	8675	DSO	1.105066
		ET	1.052632
T_Donor_PM_Blood_Gas	7875	DSO	2.197984
		ET	2.237478
T_Donor_PM_Path	3827	DSO	1.467550
		ET	1.445739
T_Donor_PM_Micro	3332	DSO	1.787917
		ET	3.046367
T_Donor_PM_Tox	1171	DSO	1.344150

Table 8:

Table	Unique Trans. Count	Data provider	Avg. Trans. Observations
T_Transplantation	14954	ET	1.000000
T_Kidney_Retrieval	14155	ET	1.000000
T_Kidney_Followup	11718	ET	1.277436
T_Postop_Exam	4768	ET	5.800336
T_Kidney_Med	2471	ET	6.609875

9 Supplementary Table 9 and 10

The following table shows baseline characteristics for selected variables of recipients, donors and transplantations in the analyzed target population. The second table describes where this information was sourced from in the TxReg data.

Table 9: Target Population Summary (selected variables)

Characteristic	Summary	Missing, n (Percent)
**Recipient**		
Age (years)	58.4 (48.3–66.0)	0 (0.0%)
Country of Origin: Germany	14945 (99.9%)	0 (0.0%)
Country of Origin: [Other Values]	9 (0.1%)	
Primary Diagnosis: [Other Values]	4881 (32.6%)	49 (0.3%)
Primary Diagnosis: Chronic renal failure - etiology uncertain	1980 (13.2%)	
Primary Diagnosis: Glomerulonephritis - histologically not examined	1824 (12.2%)	
Primary Diagnosis: Polycystic Kidneys - Adult type (dominant)	1294 (8.7%)	
Primary Diagnosis: IgA nephropathy (proven by immunofluorescence)	1207 (8.1%)	
Primary Diagnosis: Glomerulonephritis - histologically examined	1053 (7.0%)	
Primary Diagnosis: Diabetes Type II	993 (6.6%)	
Primary Diagnosis: Renal vascular disease due to hypertension	853 (5.7%)	
Primary Diagnosis: Other identified renal disorders - Specify	820 (5.5%)	
Sex: male	9504 (63.6%)	0 (0.0%)
Sex: female	5450 (36.4%)	
Waiting Time (years)	3.1 (1.1–5.5)	0 (0.0%)
**Donor**		
Age (years)	57.2 (47.1–68.1)	28 (0.2%)
Cause of Death: I60.9	3367 (22.5%)	1366 (9.1%)
Cause of Death: [Other Values]	3104 (20.8%)	
Cause of Death: I61.9	1958 (13.1%)	
Cause of Death: G93.1	1814 (12.1%)	
Cause of Death: S06.9	1623 (10.9%)	
Cause of Death: I62.9	908 (6.1%)	
Cause of Death: I63.5	814 (5.4%)	
Sex: male	7856 (52.5%)	21 (0.1%)
Sex: female	7077 (47.3%)	
**Transplantation**		
Assignment Program: ETKAS	10554 (70.6%)	11 (0.1%)
Assignment Program: ESP	4275 (28.6%)	
Assignment Program: [Other Values]	114 (0.8%)	

Note:
Continuous variables: Median (IQR); Categorical: n (Percent). Missing shown on the first level only.

Table 10: Data sources for baseline characteristics

Variable	Table	Columns	Comment	Data Provider

Donor Age (years)	T_Donor_PM	C_Age, C_BirthDate	Mean used age and birthdate to transplantation	DSO, ET, IQTIG
Donor Cause of Death	T_Donor_PM_Diag	C_DiagClass, C_DiagDescr	Type had to be "Todesursache"	DSO
Donor Sex	T_Donor_PM	C_Sex		DSO, ET, IQTIG
Recipient Age (years)	T_Recipient	C_BirthDate		ET
Recipient Country of Origin	T_Recipient	C_Country		ET
Recipient Primary Diagnosis	T_Kidney_Waitlist	C_BaseDisease, C_BaseDiseaseDescr		ET, IQTIG
Recipient Sex	T_Recipient	C_Sex		ET, IQTIG
Recipient Waiting Time (years)	T_Recip_Urgency	C_UrgencyDate	Time between first and last	ET
Transplantation Assignment Program	T_Transplantation	C_AllocationProgram		ET
